# Response mechanisms induced by exposure to high temperature in anthers from thermo-tolerant and thermo-sensitive tomato plants: A proteomic perspective

**DOI:** 10.1371/journal.pone.0201027

**Published:** 2018-07-19

**Authors:** Maria Fiorella Mazzeo, Giuseppina Cacace, Paolo Iovieno, Immacolata Massarelli, Stefania Grillo, Rosa Anna Siciliano

**Affiliations:** 1 Institute of Food Sciences—National Research Council of Italy (CNR-ISA), Avellino Italy; 2 Institute of Biosciences and Bioresources—National Research Council of Italy (CNR-IBBR), Portici, Napoli, Italy; Northeast Forestry University, CHINA

## Abstract

Constant global warming is one of the most detrimental environmental factors for agriculture causing significant losses in productivity as heat stress (HS) conditions damage plant growth and reproduction. In flowering plants such as tomato, HS has drastic repercussions on development and functionality of male reproductive organs and pollen. Response mechanisms to HS in tomato anthers and pollen have been widely investigated by transcriptomics; on the contrary, exhaustive proteomic evidences are still lacking. In this context, a differential proteomic study was performed on tomato anthers collected from two genotypes (thermo-tolerant and thermo-sensitive) to explore stress response mechanisms and identify proteins possibly associated to thermo-tolerance. Results showed that HS mainly affected energy and amino acid metabolism and nitrogen assimilation and modulated the expression of proteins involved in assuring protein quality and ROS detoxification. Moreover, proteins potentially associated to thermo-tolerant features, such as glutamine synthetase, S-adenosylmethionine synthase and polyphenol oxidase, were identified.

## Introduction

High temperature can be considered one of the most detrimental environmental factors for agriculture as it affects plant growth and reproduction thus leading to significant losses in productivity [1]. This is particularly relevant as global warming is a constant increasing phenomenon since 1900 and plants exhibiting thermo-tolerant traits, through the modulation of specific molecular mechanisms to prevent or repair heat damage, are very likely to acquire relevant agricultural value.

Effects of heat stress (HS), which include transitory or constant high temperature exposure, encompass changes in plant morphology, physiology and biochemistry involving a re-organization of cell structure and metabolism and alterations in the accumulation of several proteins and primary and secondary metabolites [2–4]. Of note, HS is also associated to oxidative stress, as HS induces the production of reactive oxygen species (ROS) such as singlet oxygen (^1^O_2_), superoxide ion (O_2_^•-^), hydrogen peroxide (H_2_O_2_), and hydroxyl radical (OH^•^) that cause remarkable damages to plant. To face off with oxidative stress, plants have developed ROS detoxification systems that include enzymatic and non-enzymatic antioxidant components [4,5]. Moreover, HS triggers the accumulation of other compounds such as proline, glycine betaine, sugars and phenols that play a protective role versus cell membranes and ROS action. In addition, HS affects respiration, photosynthesis and membrane fluidity also associated to the presence of ROS [2,6]. In flowering plants such as rice, barley and tomato, the anther and pollen development is particularly affected by exposure to high temperatures, causing the loss of fruit set [7–9].

The impact of HS on male reproductive organs and pollen has been extensively studied at transcriptomic level leading to the identification of genes regulated by HS and involved in crucial metabolic processes [10–14]. In particular, a key role of heat shock proteins (Hsps) and heat stress transcription factors (HSfs) has been depicted [15,16].

In addition, several proteomic studies have contributed to clarify organ-specific response mechanisms to abiotic and biotic stress conditions in crop species [17–19], although only a few of them have investigated HS response in anthers and pollen of flowering plants [20,21]. Pioneering studies reported first information on the effect of cold stress on maturation of rice anthers [22,23]. More recently, heat shock proteins that might contribute to heat tolerance at anthesis in the tolerant cv. N22 rice genotype were identified [24]. The over expression of heat shock proteins as well as β-expansins and lipid transfer proteins in this resistant cultivar was also reported [25]. Similarly, shotgun proteomic experiments showed that the exposure to high temperatures increased the expression of heat shock proteins and trehalose synthase proteins in rice anthers from a high temperature tolerant Japonica rice variety Dianxi4, suggesting a key role of these proteins in conferring tolerance to rice anthers [26]. Response to salt stress and water deficit has also been studied in rice anthers by proteomics [27,28]. Proteomics has been also applied to provide information on molecular mechanisms involved in the induction of microspore embryogenesis in maize by cold pre-treatment [29].

More recently, the release of the complete genome sequence of tomato [30] prompted proteomic studies focused on the identification of proteins involved in abiotic stress response in several tissues and organs [31]. However, studies on the anther tomato proteome are still very scarce. Sheoran and colleagues used a proteomic approach to reveal changes in protein expression profiles of anthers from a male-sterile mutant of tomato compared to the wild type and discussed the functional role of these changes in anther and pollen development and in male-sterility [32]. It is worth to note that the HS response and tolerance mechanisms in tomato anthers have not been investigated at protein level yet.

Therefore, in the present study, proteomic analyses were performed on tomato anthers collected from flowers of *Solanum lycopersicum* cv M82 and cv Saladette (SAL). M82 cultivar is considered a tomato model genotype widely used in experimental studies. It is sensitive to HS occurring during the reproductive developmental phase including flower development and fruit set, as most of the cultivated tomato varieties. On the other hand, SAL is reported to be as one of the most thermo-tolerant genotype [33,34]. This study was aimed to elucidate molecular mechanisms underlying the plant response to high temperature growth condition and identify proteins constitutively expressed at higher or lower level in the tolerant genotype, thus defining processes involved in the efficient reduction of the adverse effects of HS. Results showed differences in the abundance of ninety-six proteins and their functional classification highlighted that heat stress mainly affected metabolic pathways, such as energy metabolism (glycolysis and sucrose degradation), nitrogen assimilation and amino acid biosynthesis and modulated the expression of proteins involved in the folding and degradation machinery and ROS detoxification systems.

This study could contribute to clarify the physiological response of tomato to high temperature and molecular mechanisms of heat tolerance, which is a central point in designing ad hoc strategies to improve crop thermo-tolerance.

## Materials and methods

### Plant growth, heat stress conditions and sample collection

Plants of *Solanum lycopersicum* thermo-tolerant (cv Saladette, SAL) and thermo-sensitive (M82) genotypes were grown in greenhouse under controlled temperature conditions (CC) (26°C/20°C day/night) and natural illumination. After a month, plants designated for the HS treatment were moved in a different greenhouse and subjected to high temperature conditions (HT) (36°C\25°C (day\night) under natural illumination). These growth conditions were kept for a total period of three months in which developing flower buds of different stages corresponding to the morphological stages described by Brukhin and colleagues [35] were harvested continuously from both genotypes.

Flower buds (7–8 mm) from 25 plants for each genotype grown under CC and HT conditions from the first or second truss were collected. Tomato buds of 8 mm in length show unambiguous morphological features (such as constant dimensions within the genotypes, semi-open sepals and white-coloured corolla) and their physiological stage is about 7 days before the anthesis (Massarelli and Grillo, unpublished). Two biological replicates were prepared and each replica consisted of about 150 flower buds, which were separated in different tissues (sepals, petals and anther tissues), pooled in aliquots, quickly frozen and stored at -80°C. The anther samples were used for proteomic analyses after a careful removal of pollen grains present in this physiological phase [35].

### Proteome extraction

The anthers collected from 7-8mm flower buds were grinded to a fine powder with pestle and mortar in liquid nitrogen. Proteome extraction was carried out on this powder by a phenol extraction followed by methanolic ammonium acetate precipitation according to the protocol of Hurkman and Tanaka [36]. In brief, 0.8 g of anthers were treated with 2 mL Tris-HCl pH 8.0 buffered phenol and 2 mL of extraction media (0.1 M Tris-HCl pH 8.0, 10 mM EDTA, 0.4% 2-mercaptoethanol, 0.9 M sucrose). Phenol extracted proteins were precipitated by adding 10 volumes of 0.1 M ammonium acetate in methanol (pre-chilled to -80°C) to the phenol phase at -80°C overnight. Protein pellets, collected by centrifugation, were dissolved in 200 μL buffer solution (8 M Urea, 4% Chaps, 40 mM Tris-HCl, 40 mM DTT) and protein concentration was determined using the Bradford protein assay (Bio-Rad, Hercules, CA, USA). All reagents and solvents used in this study were of the highest purity and purchased from Sigma-Aldrich (Saint Louis, MO, USA).

### 2-DE and image analysis

2-DE and image analysis were performed as already described [37]. Briefly, IEF was performed using the Ettan IPGphor (GE Healthcare, Amersham Biosciences AB, Uppsala, Sweden), whilst the SDS-PAGE was carried out using the MiniProtean (Bio-Rad). 300 μg protein samples were applied by in-gel rehydratation (according to the manufacturer’s instructions) in 7-cm IPG strips, pH 4–7. Protein spots were visualized by staining with Coomassie Brilliant Blue G-250. Protein extracts obtained, for each sample, from the two biological replicates, were run in duplicate. Therefore, each sample was run in quadruplicate for a total of 16 gel maps (S1 Fig). 2-DE protein patterns were recorded as digitalized images using a high-resolution scanner (GS-710 Calibrated Imaging Densitometer, Bio-Rad). Spot detection, quantization, and analysis were performed using the PDQuest™ 2-D Analysis Software, Version 6.2 (Bio-Rad).

Four different comparisons were carried out, in particular Image Analysis I (IA I) was the comparison between the 2-DE maps obtained from the analysis of the proteomes extracted from SAL anthers grown under HT and CC and Image Analysis II (IA II) was the same analysis referred to M82. Image Analysis III (IA III) and Image Analysis IV (IA IV) indicated the comparison between the 2-DE maps obtained from the analysis of SAL (control sample) and M82 proteomes extracted from anthers grown under the same condition, (CC and HT, respectively).

Spots whose mean intensity showed a 2-fold or higher change in at least one of the image analyses and having a Student's t-test confidence level of 0.05 were chosen for mass spectrometric analyses. A fold change higher that 1.5 was considered biologically relevant. Mean normalized spot volume and standard deviation (SD) were determined for each spot (S1 Table).

### Protein identification

Spots were excised from 2-DE gels and in-gel tryptic digestion was carried out. Protein identification was achieved by peptide mass fingerprint (PMF) strategy and nanoESI-LC-MS/MS experiments. Samples, desalted using μZipTipC18 tips according to manufacturer protocols (Millipore, Billerica, MA, USA), were analyzed on a M@LDI mass spectrometer (Waters, Milford, MA, USA) operating in positive-ion reflectron mode. Mass spectra were processed using the MassLynx 2.1 and ProteinLynx Global Server software (Waters). Peak lists were manually inspected. Protein identification was achieved by using peak lists for searches against the NCBInr database using the Mascot algorithm (http://www.matrixscience.com/). Parameters for all searches were as follows: all entries as taxonomic category, trypsin as enzyme, carbamidomethyl as fixed modification for cysteine residues and methionine oxidation as variable modification, up to two missing cleavages and up to 50 ppm as mass tolerance [37].

NanoESI-HPLC-MS/MS experiments were carried out on a Q-TOF Micro instrument equipped with a nanoelectrospray Z spray source and a capillary flow-liquid chromatography system (CapLC, Waters). Peptide samples were loaded, purified and concentrated on a pre-column Symmetry300 C18 Trap Column, 0.18 x 23 mm, 5 μm (Waters) and separated on a nano column Atlantis dC18, 75 μm x 150 mm, 3 μm (Waters). Data Dependent Acquisition (DDA) was carried out as already described [37]. MS/MS data were used for achieving protein identification by querying the NCBInr database with the Mascot algorithm option MS/MS Ion Search. Parameters for all searches were the same previously reported except for peptide mass tolerance (0.3 Da) fragment mass tolerance (0.2 Da) and taxonomic category (viridiplantae (green plants)).

### Bioinformatics

Identified proteins were classified by means of the bioinformatic resource SolCyc Biochemical Pathways (http://solcyc.solgenomics.net/) using as organism database *Solanum lycopersicum* to define biological functions and metabolic pathways involved in HS response. Hierarchical cluster analysis of differentially represented proteins in the four Image Analyses was performed using Genesis 1.7.7 software (http://genome.tugraz.at/genesisclient/genesisclient_description.shtml). Protein interaction networks were obtained with STRING (http://string-db.org). Active prediction methods used in our analysis were neighbourhood, coexpression, experiments, co-occurrence, databases and text mining, using custom confidence value of 0.600.

The mass spectrometry proteomics data have been deposited to the ProteomeXchange Consortium via the PRIDE (www.proteomexchange.org) partner repository with the dataset identifier PXD010156.

## Results

Heat stress response mechanisms were investigated in tomato anthers collected from flowers SAL and M82 genotypes. Anthers from 7–8 mm flower buds were selected as microgametogenesis occurs in this physiological stage [12], which is also highly sensitive to HS [38].

Pollen viability was measured to evaluate the impact of HT condition on the two tomato genotypes. Results on this physio-agronomic parameter further confirmed the thermo-tolerance features of SAL as its pollen viability decreased of 9% under HT while M82 pollen viability decreased of 25%. (S1 Appendix).

**Fig 1:**
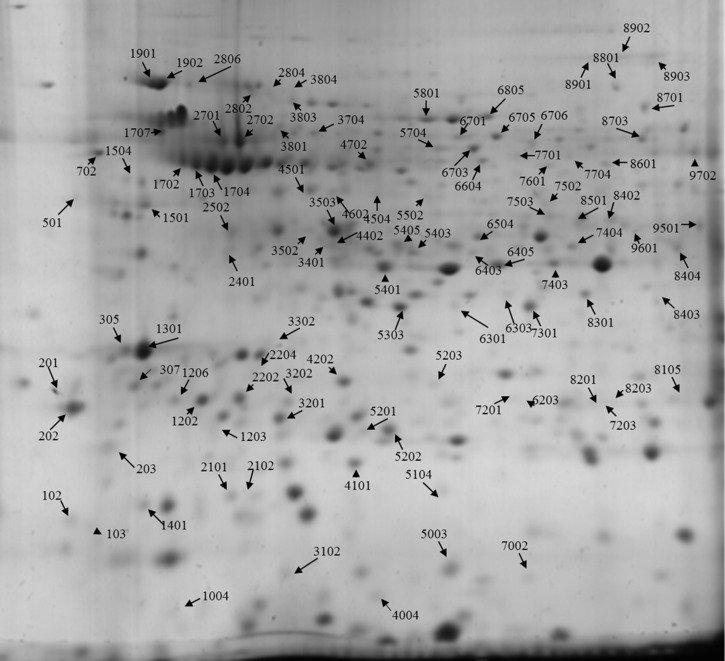
Representative 2-DE gel of anther proteome. The proteomic map of M82 under CC is reported. Spots exhibiting significant differences in mean volume are indicated.

In order to highlight biochemical processes affected by HT in the two genotypes, a proteomic study was performed. Image analysis led to detect 106 spots whose relative intensities varied in the 2-DE maps obtained from the analysis of the proteomes extracted from SAL and M82 anthers grown under CC and HT (Fig 1). In seven cases, the analysis of adjacent spots in the 2-DE maps led to the identification of the same protein, thus indicating the presence of isoforms probably due to post-translational modifications (S2 Table). Therefore, 98 proteins were identified and two of them were not included in the further functional analyses as they co-migrated in spot 4702 (S2 and S3 Tables).

**Fig 2:**
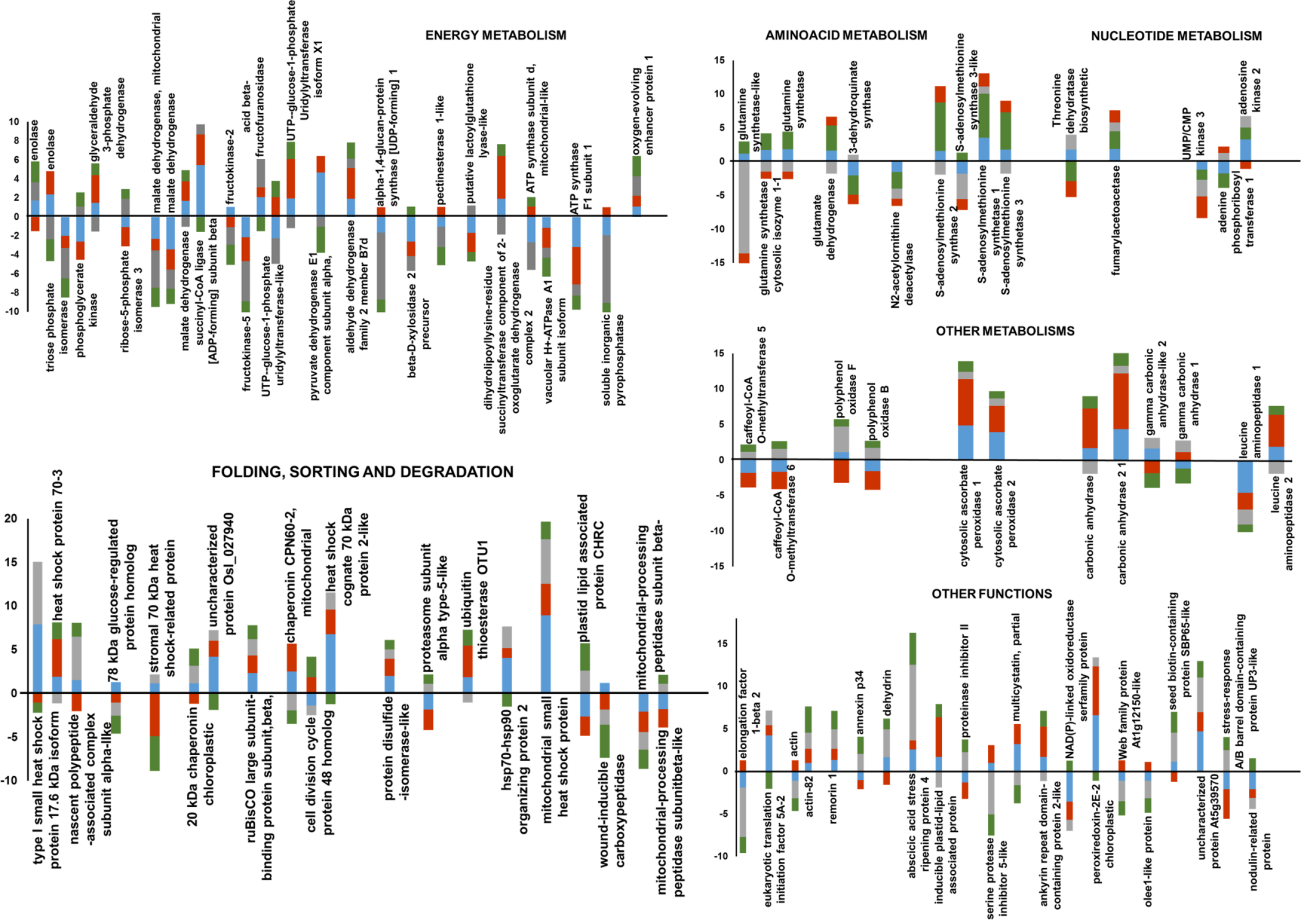
Bar diagram summarizing changes in abundance of differentially modulated proteins. These proteins are grouped based on their functional classification.

**Fig 3:**
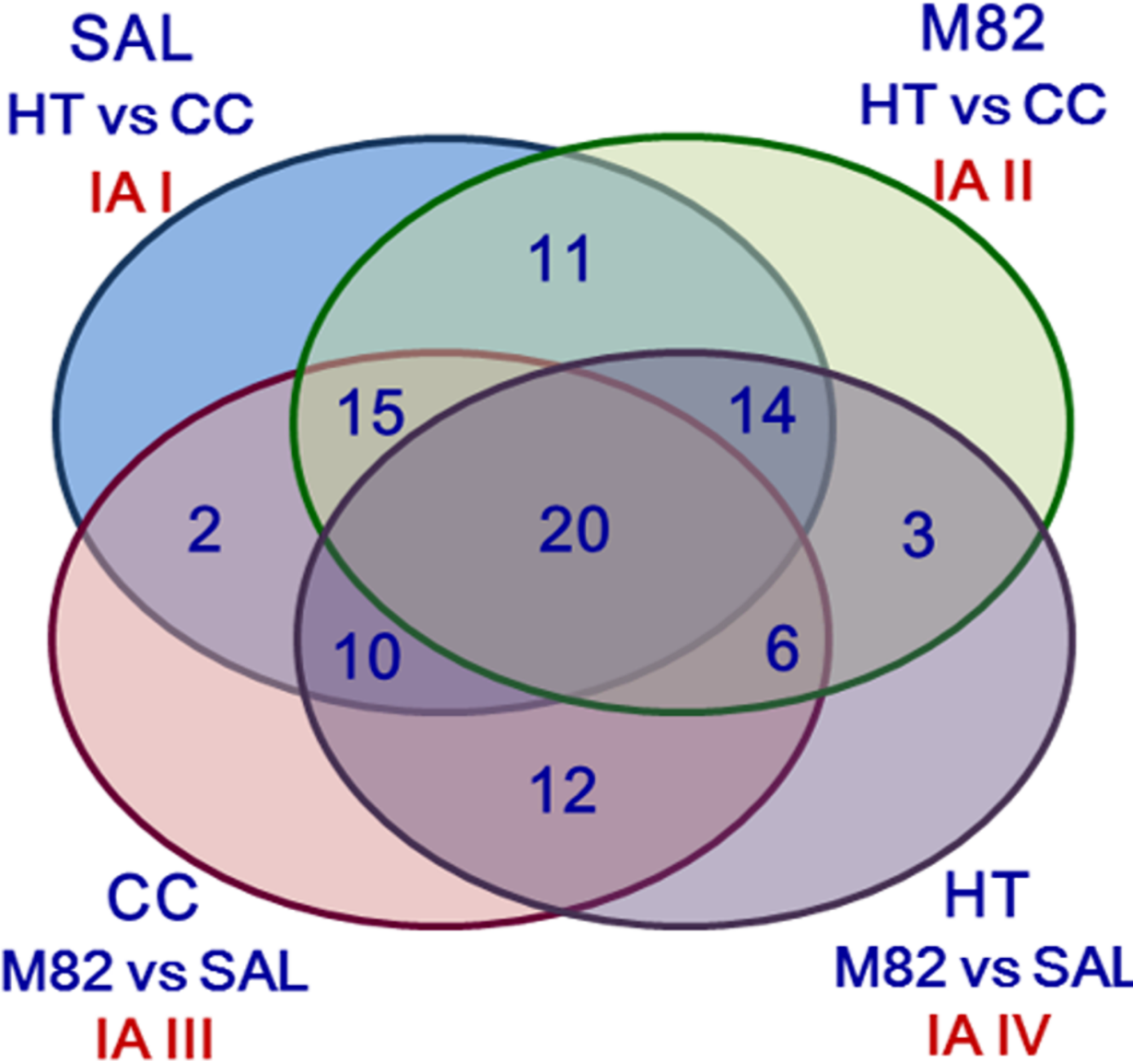
Venn diagram summarizing proteomic results. The number of proteins showing significant changes in abundance in the four Image Analyses is reported. Proteins changing their abundance in both Image Analyses I and IV and in both Image Analyses II and III (one protein and two proteins, respectively) are not included in the diagram (see Table 1).

The functional classification of the identified proteins and the image analyses data are reported in Table 1 and Fig 2 and proteomic results have been summarized in the Venn diagram reported in Fig 3. One protein changed its expression level in Image Analysis I and Image Analysis IV and two proteins changed their expression level in Image Analysis II and Image Analysis III and they could not be represented in the Venn diagram.

**Table 1:** Proteins differentially represented in SAL and M82 anther proteomes. Functional classification and regulation of protein abundance are reported.

				HT/CC (fold change) ^a)^		M82/SAL (fold change) ^b)^	
ACCESSION NCBInr	PROTEIN NAME	SOL GENOMICS NETWORK ID GENE ID	PATHWAY OR GO TERMS	IA I SAL	IA II M82	IA III CC	IA IV HT
			**ENERGY METABOLISM**				
XP_004243619.1	2,3-bisphosphoglycerate-independent phosphoglycerate mutase	Solyc07g044840.2 101248497	Glycolysis I-V(Plant Cytosol)	-3.32	-2.12	-2.04	-1.30
NP_001332774.1	enolase	Solyc10g085550.1 101247482		1.72	-1.50	1.91	2.21
NP_001234080.1	enolase	Solyc09g009020.2 544068 PGH1, ER28		2.38	2.44	-2.35	-2.30
XP_004230885.1	triose phosphate isomerase chloroplastic	Solyc01g111120.2.1101246286		-2.01	-1.26	-3.21	-2.01
NP_001316521.1	phosphoglycerate kinase	Solyc07g066600.2 101254111		-2.59	-1.93	1.08	1.50
NP_001266254.2	glyceraldehyde 3-phosphate dehydrogenase	Solyc05g014470.2 101258368 GAPC2, GAPDH		1.50	2.88	-1.55	1.24
XP_004230104.1	probable ribose-5-phosphate isomerase 3, chloroplastic	Solyc01g097460.2 101264255	Rubisco Shunt	-1.06	-2.02	1.92	1.01
NP_001315977.1	malate dehydrogenase, mitochondrial	Solyc12g014180.1 101258530 mMDH2	TCA Cycle Variation III-IV Glyoxylate Cycle	-2.32^c^	-1.20 ^c^	-3.94 ^c^	-2.04 ^c^
NP_001234001.2	mitochondrial malate dehydrogenase	Solyc07g062650.2 7782185 mMDH		-3.41	-2.14	-2.01	-1.62
XP_004247734.1	malate dehydrogenase	Solyc09g090140.2 101253131		1.71	2.05	-1.03	1.17
NP_001234293.2	succinyl-CoA ligase [ADP-forming] subunit beta, mitochondrial	Solyc06g083790.2 543863		5.44	3.26	1.06	-1.57
NP_001233888.1	fructokinase-2	Solyc06g073190.2 544022 FRK2	Sucrose Degradation III-I UDP-Galactose Biosynthesis	1.07	-1.06	-1.87	-2.13
XP_010313061.1	probable fructokinase-5	Solyc11g042850.1 101261091		-2.13	-2.54	-4.21	-5.02
NP_001234843.2	acid beta-fructofuranosidase precursor	Solyc03g083910.2 543992 AI Aiv-1, TAI, TIV1		2.07	1.03	2.99	-1.50
XP_004239832.1	UTP—glucose-1-phosphate uridylyltransferase-like	Solyc05g054060.2 101250892		-2.23	2.08	-2.70	1.72
XP_004250240.1	UTP—glucose-1-phosphate uridylyltransferase isoform X1	Solyc11g011960.1 101248935		1.94	4.15	-1.19	1.81
NP_001296789.1	pyruvate dehydrogenase E1 component subunit alpha, mitochondrial	Solyc05g006520.2 543639	Acetyl-Coa Biosynthesis	4.66	1.74	-1.03	-2.74
NP_001333533.1	aldehyde dehydrogenase family 2 member B7d	Solyc05g005700.2 101254485 SlADH2B7d	Dopamine Degradation Oxidative Ethanol Degradation III-I	1.91	3.21	1.01	1.69
XP_004232138.1	alpha-1,4-glucan-protein synthase [UDP-forming] 1-like	Solyc02g089170.2 101251942	Cellulose Biosynthesis	-1.64	1.01	-7.07	-4.25
NP_001296718.1	beta-D-xylosidase 2 precursor	Solyc01g104950.2 543515	Xylan 1,4-Beta-Xylosidase Activity	-2.61	-1.51	-1.58	1.10
XP_004239981.3	pectinesterase 1-like	Solyc05g052120.2 101243787	Homogalacturonan Degradation	-1.02	1.07	-2.13	-1.94
XP_004232705.1	putative lactoylglutathione lyase-like	Solyc02g080630.2 101251054	Methylglyoxal Degradation I	-1.69	-2.01	1.19	-1.01
XP_004244101.1	dihydrolipoyllysine-residue succinyltransferase component of 2-oxoglutarate dehydrogenase complex 2, mitochondrial-like	Solyc07g064800.2 101268590	2-Ketoglutarate Dehydrogenase Complex	1.90	4.50	-1.87	1.26
XP_004251263.1	ATP synthase subunit d, mitochondrial-like	Solyc11g072450.1 101248453	ATP Synthesis Coupled Proton Transport	-2.66	1.09	-2.91	1.00
NP_001234281.2	vacuolar H+-ATPase A1 subunit isoform	Solyc12g055800.1 543861	ATP Hydrolysis Coupled Proton Transport	-1.13	-2.09	-1.08	-2.00
YP_009430460.1	ATP synthase F1 subunit 1	Solyc11g039980.1 34678293atp1	Proton-Transporting Two-Sector Atpase Complex	-3.15	-3.95	-1.18	-1.50
XP_004252616.1	soluble inorganic pyrophosphatase PPA1	Solyc08g083370.2 101249338	Phosphate-Containing Compound Metabolic Process	-1.92	1.03	-7.15	-3.61
NP_001296294.1	Oxygen-evolving enhancer protein 1, chloroplastic	Solyc02g065400.2 544299 PSBO	Photosynthesis	1.10	1.13	2.07	2.12
			**AMINOACID METABOLISM**				
XP_004240034.1	glutamine synthetase-like	Solyc05g051250.2 101261030	Nitrate Reduction II-VI (Assimilatory) Ammonia Assimilation Cycle II Glutamine Biosynthesis	1.16	1.83	-13.59	-8.58
NP_001309987.1	glutamine synthetase cytosolic isozyme 1–1	Solyc04g014510.2 543756 gts1		1.75	2.44	-1.50	-1.04
NP_001310599.1	glutamine synthetase	Solyc01g080280.2 543998 GS2		1.86	2.59	-1.50	-1.06
NP_001292722.1	glutamate dehydrogenase	Solyc10g078550.1 544015 gdh1	Glutamate Biosynthesis/ Degradation	1.62	3.74	-1.78	1.30
NP_001233863.1	3-dehydroquinate synthase, chloroplastic	Solyc02g083590.2 544273 DHQS		-2.05	-2.86	1.01	-1.39
NP_001316163.1	N2-acetylornithine deacetylase	Solyc08g076970.2 101268129 NAOD	Arginine Biosynthesis III Ornithine Biosynthesis	-1.55	-2.43	-1.51	-1.04
NP_001296305.1	S-adenosylmethionine synthase 2	Solyc12g099000.1 101247506 SAM2	Phytosiderophore Biosynthesis S-Adenosyl-L-Methionine Cycle/Biosynthesis	1.56	7.21	-1.94	2.38
XP_010312254.1	S-adenosylmethionine synthase 3-like	Solyc10g083970.1 101245012		-1.77	1.31	-3.76	-1.62
NP_001234425.1	S-adenosylmethionine synthetase 1	Solyc01g101060.2 544155 SAM1		3.55	6.52	1.06	1.95
NP_001234004.1	S-adenosylmethionine synthetase 3	Solyc09g008280.1 544302 SAM3		1.81	5.49	-1.77	1.71
NP_001296095.1	Threonine dehydratase biosynthetic, chloroplastic	Solyc09g008670.2 543983	Isoleucine Biosynthesis Hypoglycin Biosynthesis	1.81 ^c^	-2.86 ^c^	2.17 ^c^	-2.38 ^c^
XP_019068856.1	fumarylacetoacetase	Solyc04g014730.2 101261704	Tyrosine Degradation I	1.87	2.61	1.31	1.82
			**OTHER METABOLISMS**				
XP_004248757.1	caffeoyl-CoA O-methyltransferase 5	Solyc10g050160.1 101253032	Phenylpropanoid Biosynthesis	-1.80	-2.05	1.17	1.03
XP_004230766.1	caffeoyl-CoA O-methyltransferase 6	Solyc01g107910.2 101260278		-1.64	-2.48	1.59	1.05
NP_001318059.1	polyphenol oxidase F, chloroplastic	Solyc08g074630.1 101259064	Esculetin Biosynthesis	1.09	-3.22	3.63	1.03
NP_001296326.1	polyphenol oxidase B, chloroplastic;	Solyc08g074680.2 101258774 PPO		-1.55	-2.62	1.70	1.01
NP_001234782.1	cytosolic ascorbate peroxidase 1	Solyc06g005160.2 778223 APX1	Ascorbate Glutathione Cycle	4.86	6.50	1.06	1.50
NP_001234788.2	cytosolic ascorbate peroxidase 2	Solyc06g005150.2 778224 APX2		3.91	3.71	1.06	1.01
NP_001233847.1	carbonic anhydrase	Solyc02g067750.2 100147727 ca3	Cyanate Degradation	1.68	5.58	-1.92	1.73
NP_001296993.1	carbonic anhydrase, 2 1	Solyc05g005490.2 543802 Ca1		4.34	7.85	1.06	1.92
XP_004234310.1	gamma carbonic anhydrase-like 2, mitochondrial-like	Solyc03g019720.2 101249243		1.62	-1.83	1.50	-2.06
XP_004232424.1	gamma carbonic anhydrase 1, mitochondrial-like	Solyc02g085040.2 101264478		-1.17	1.13	1.61	-2.14
NP_001233862.2	leucine aminopeptidase 1, chloroplastic	Solyc12g010040.1 544017 LAPA1	Seed Germination Protein Turnover Wound-Induced Proteolysis I	-4.62 ^c)^	-2.33 ^c)^	-2.14 ^c)^	-1.08 ^c)^
NP_001233884.2	Leucine aminopeptidase 2, chloroplastic	Solyc00g187050.2 544277 LAPA2		1.86	4.50	-1.92	1.26
			**NUCLETOTIDE METABOLISM**				
NP_001296740.1	UMP/CMP kinase 3	Solyc01g088480.2 101243930 UMP-CMP kinase 3	Adenosine Nucleotides De Novo Biosynthesis	-1.16	-1.52	-2.46	-3.21
NP_001316139.1	adenine phosphoribosyltransferase 1	Solyc04g077970.2 101260722	Salvage Pathways Of Purine Nucleosides II	-1.72	-2.15	1.26	1.00
XP_004249586.1	adenosine kinase 2	Solyc10g086190.1 101251530		3.29	1.74	1.75	-1.08
			**FOLDING, SORTING AND DEGRADATION**				
NP_001234225.1	type I small heat shock protein 17.6 kDa isoform	Solyc06g076560.1 543848	Unfolded Protein Binding	7.93	-1.04	7.16	-1.15
NP_001266213.1	heat shock protein 70–3	Solyc04g011440.2 101055596 hsc70.3		1.94	4.27	-1.14	1.92
XP_004249331.1	nascent polypeptide-associated complex subunit alpha-like protein-like	Solyc10g081030.1 101251235		1.54	-2.04	4.97	1.58
P49118.1	78 kDa glucose-regulated protein homolog	Solyc08g082820.2 543957 BiP/grp78	Misfolded Protein Binding	1.32	-1.04	-1.50	-2.04
XP_004230445.1	stromal 70 kDa heat shock-related protein, chloroplastic-like	Solyc01g103450.2 101265681	Protein Folding	1.15 ^c)^	-4.89 ^c)^	1.06 ^c)^	-3.98 ^c)^
XP_004251703.1	20 kDa chaperonin, chloroplastic	Solyc12g009250.1 101253560	Chaperone Binding	1.16	-1.16	2.01	2.00
XP_004247428.1	uncharacterized protein OsI_027940	Solyc09g075010.2 101249490		4.22	1.80	1.25	-1.88
XP_004228946.1	ruBisCO large subunit-binding protein subunit beta, chloroplastic	Solyc01g028810.2 101253117	Cellular Protein Metabolic Process- Protein Folding	2.33	2.02	1.87	1.62
XP_004247810.1	chaperonin CPN60-2, mitochondrial	Solyc09g091180.2 101250927	ATP Binding	2.53	3.18	-1.94	-1.54
NP_001315608.1	cell division cycle protein 48 homolog	Solyc06g074980.2 101266129		-1.38	1.87	-1.10	2.35
XP_004250958.1	heat shock cognate 70 kDa protein 2-like	Solyc11g066060.1 101254866 hsp70-2		6.79	2.81	1.95	-1.24
XP_004240392.1	protein disulfide-isomerase-like	Solyc06g005940.2 101262921	Protein Disulfide Isomerase Activity	2.03	1.94	1.10	1.06
XP_004244803.1	proteasome subunit alpha type-5-like	Solyc08g016510.2 101266742	Endopeptidase Activity	-1.83	-2.36	1.14	1.07
XP_004244120.1	ubiquitin thioesterase OTU1	Solyc07g064590.2 101248409	Ubiquitin-Specific Protease Activity	1.88	3.58	-1.03	1.85
XP_004245731.1	hsp70-Hsp90 organizing protein 2	Solyc08g079170.2 101245387	Protein Kinase Inhibitor Activity	4.06	1.13	2.47	-1.50
NP_001233872.1	mitochondrial small heat shock protein	Solyc08g078700.2 543507 MTSHP		8.96	3.58	5.12	2.04
NP_001234183.1	plastid lipid associated protein CHRC	Solyc02g081170.2 778336 ChrC	Protein Binding	-2.62 ^c)^	-2.21 ^c)^	2.63 ^c)^	3.11 ^c)^
NP_001234691.2	wound-inducible carboxypeptidase	Solyc06g083040.2 544223		1.21	-1.85	-1.70	-3.82
XP_015064515.1	probable mitochondrial-processing peptidase subunit beta	Solyc02g088700.2 107009685	Protein Complex Binding	-2.08 ^c)^	-2.31^c)^	-2.01^c)^	-2.24 ^c)^
XP_004239065.1	probable mitochondrial-processing peptidase subunit beta-like	Solyc05g012480.2 101247218		-1.77	-2.10	1.10	1.07
			**OTHER FUNCTIONS**				
XP_004251245.1	elongation factor 1-beta 2	Solyc11g072190.1 101268350	Translation Elongation Factor 1 Complex	-1.89	1.30	-5.80	-1.90
NP_001234503.1	Eukaryotic translation initiation factor 5A-2	Solyc07g005560.2 543668 eIF-5A2	Protein N-Terminus Binding	4.24	1.18	1.76	-2.03
NP_001317048.1	actin -41	Solyc04g011500.2 101260631 actin	Structural Constituent Of Cytoskeleton	-1.08	1.30	-2.06	-1.50
XP_004241231.1	actin-82	Solyc06g076090.2 101249734 actin-82		1.00	1.67	1.88	3.07
NP_001234231.1	remorin 1	Solyc03g025850.2 543593 rem-1	DNA Binding	1.35	1.29	2.30	2.19
NP_001234104.1	annexin p34	Solyc04g073990.2 543564 AN34	Calcium-Dependent Phospholipid Binding	-1.04	-1.06	2.06	2.02
NP_001316365.1	dehydrin	Solyc04g082200.2 101253585 dhn	Response To Water	1.65	-1.59	3.31	1.26
NP_001269248.1	abscicic acid stress ripening protein 4	Solyc04g071620.2 101246420 Asr4	Response To Stress	2.56	1.07	8.92	3.73
NP_001266269.1	inducible plastid-lipid associated protein	Solyc07g064600.2 101248695 CHRDi	Endoribonuclease Activity	1.72	4.67	-1.89	1.53
AFJ93093.1	proteinase inhibitor II	Solyc03g020060.2	Serine-Type Endopeptidase Inhibitor Activity	-1.28	-1.94	2.28	1.50
XP_004235415.1	serine protease inhibitor 5-like	Solyc03g098760.1 101263388	Endopeptidase Inhibitor Activity	1.01	2.07	-5.06	-2.47
XP_004253396.2	multicystatin, partial	Solyc00g071180.2 543570	Cysteine-Type Endopeptidase Inhibitor Activity Cobalt Ion Binding	3.21 ^c)^	2.39 ^c)^	-1.60 ^c)^	-2.16 ^c)^
XP_004230497.1	ankyrin repeat domain-containing protein 2-like	Solyc01g104170.2 101255143	Mechanically-Gated Ion Channel Activity	1.71	3.57	-1.12	1.86
NP_001317632.1	NAD(P)-linked oxidoreductase serfamily protein	Solyc07g043570.2 101252603	Oxidation-Reduction Process	-3.54	-2.13	-1.30	1.27
XP_010312248.1	peroxiredoxin-2E-2, chloroplastic	Solyc10g083650.1 104644379	Oxidoreductase Activity	6.61	5.74	1.06	-1.09
XP_004237119.2	Web family protein At1g12150-like	Solyc04g015110.2 101255879		-1.10	1.33	-2.41	-1.65
XP_015069432.1	pollen allergen Che a 1-like	107014082		-1.11	1.10	-2.05	-1.68
XP_004243405.2	seed biotin-containing protein SBP65-like	Solyc07g053360.2 101259649		1.15	-1.21	3.40	2.45
XP_004232206.1	uncharacterized protein At5g39570	Solyc02g088260.2 101248442		4.71	2.24	4.08	1.94
XP_010324012.1	stress-response A/B barrel domain-containing protein UP3-like	Solyc07g041490.1 101256396		-2.10	-3.47	2.50	1.52
XP_004239074.1	nodulin-related protein	Solyc01g104780.2 101249727		-2.08	-1.04	-1.31	1.54

a) Changes in protein levels are reported as the ratio between the normalized protein spot volume from Saladette and M82 tomato anthers grown under high temperature and control conditions (V_HT_/V_CC_) for up-regulated proteins and as the negative reciprocal values (-V_HT_/V_CC_) for down-regulated proteins.

b) Changes in protein levels are reported as the ratio between the normalized protein spot volume from M82 and Saladette tomato anthers grown under the same conditions (V_M82_/V_SAL_) for up-regulated proteins and as the negative reciprocal values (- V_M82_ /V_SAL_) for down-regulated proteins.

c) Average fold change for proteins contained in more than one spot has been calculated summing the normalized protein spot volume of all the spots containing the same protein and a fold change ≥ 2.0 has been considered significant.

**Fig 4:**
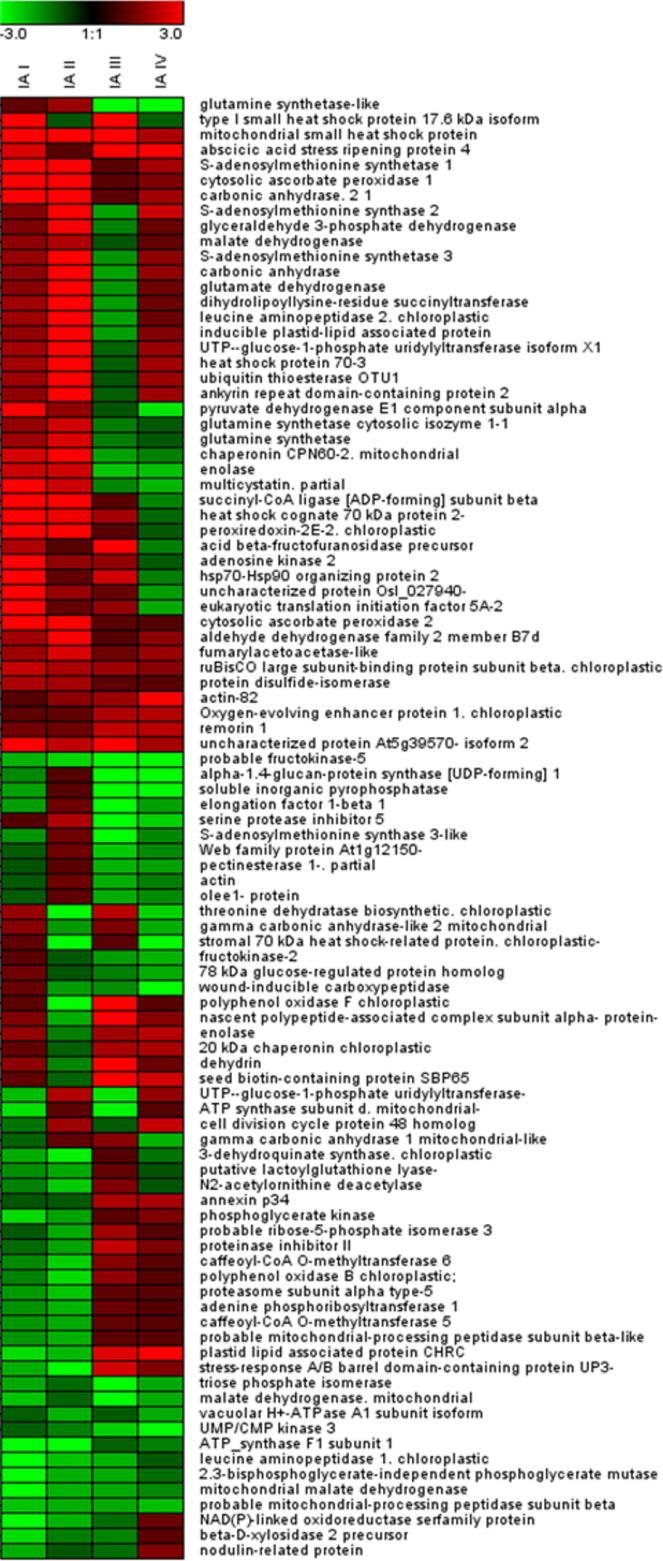
Heat-map representation of the identified proteins. Fold change values in the four Image Analyses are reported according to a color-based representation.

The two genotypes exhibited similar response mechanisms to high temperature conditions imposed during plants growth by modulating the expression level of sixty proteins in both genotypes (more than 60% of all the identified proteins) (Fig 3). Interestingly, fifty-four of these proteins showed the same trend of regulation, as the amount of thirty-five of them increased and that of the other nineteen decreased in both genotypes, as also evident in the heat map representation (Table 1, Fig 4). Only the expression of six proteins was dissimilarly influenced by high temperature: five proteins were up-regulated in the tolerant genotype SAL and down-regulated in sensitive M82, and one protein showed exactly the opposite trend.

Moreover, the expression of twelve proteins was modulated by HT only in SAL (Fig 3). In fact, small heat shock protein 17.6 KDa was highly induced by HT and present in lower amount in SAL compared to M82 under CC. These results were also confirmed by determining the relative expression level of *Hsp17*.*6* evaluated by qRT-PCR. In particular, qRT-PCR evidenced that HT induced a significant increase of *Hsp 17*.*6* transcript level only in SAL. Furthermore, a direct comparison between the two genotypes highlighted that, under CC, *Hsp 17*.*6* transcript level was higher in M82 than in SAL and similar transcript levels were present in the two genotypes under HT (S2 Appendix).

Contrarily, mitochondrial ATP synthase subunit d was expressed in lower amount under HT and present in higher amount in SAL compared to M82 under CC (Image Analyses I and III) (Table 1, Fig 4). Interestingly, six proteins whose expression was decreased by HT were more abundant in SAL compared to M82 under both growth conditions. The expression of abscicic acid stress ripening protein 4 was induced by HT and it was less abundant in SAL compared to M82 under both growth conditions. HT triggered in SAL the expression of other three proteins (acid beta-fructofuranosidase precursor, eukaryotic translation initiation factor 5A-2, hsp70-Hsp90 organizing protein 2) that were more abundant in this genotype compared to M82 under CC and less abundant under HT (Image Analyses I, III, IV) (Table 1, Fig 4).

On the other hand, nine proteins changed their expression level only in M82 grown under HT (Fig 3). In particular, the expression of vacuolar H+-ATPase A1 subunit isoform and stromal 70 kDa heat shock-related protein, present at lower level in this genotype under HT compared to SAL, was down-regulated (Image Analyses II and IV). On the contrary, cell division cycle protein 48 homolog exhibited an opposite regulation in both Image Analyses. In addition, HT induced the expression of glutamine synthetase-like and serine protease inhibitor 5-like, while reduced that of UMP/CMP kinase 3 and wound-inducible carboxypeptidase; these four proteins were less abundant in M82compared to SAL under both growth conditions. The expression of proteinase inhibitor II and actin-82 were respectively down-regulated and up-regulated by HT in the sensitive genotype and these proteins were more abundant in M82 compared to SAL under both growth conditions (Analysis II, III and IV) (Table 1, Fig 4).

Finally, the comparison between the 2-DE maps obtained from the analysis of SAL and M82 proteomes extracted from anthers grown under the same conditions (CC and HT) highlighted that the expression level of forty-eight proteins was affected by the genotype under both CC and HT. The abundance of thirty-four proteins was modulated in the two genotypes under CC or under HT, while only the amount of twelve proteins was not affected by the growth under HT and could be associated to specific features of the two varieties (Fig 3). Eleven proteins showed the same trend of regulation, as six of them were less abundant and five more abundant in the sensitive genotype in both growth conditions. One protein (mitochondrial gamma carbonic anhydrase 1) showed a different regulation, being present in higher and lower amount in M82 under CC and HT, respectively (Image Analysis III and Image Analysis IV.) (Table 1, Fig 4).

**Fig 5:**
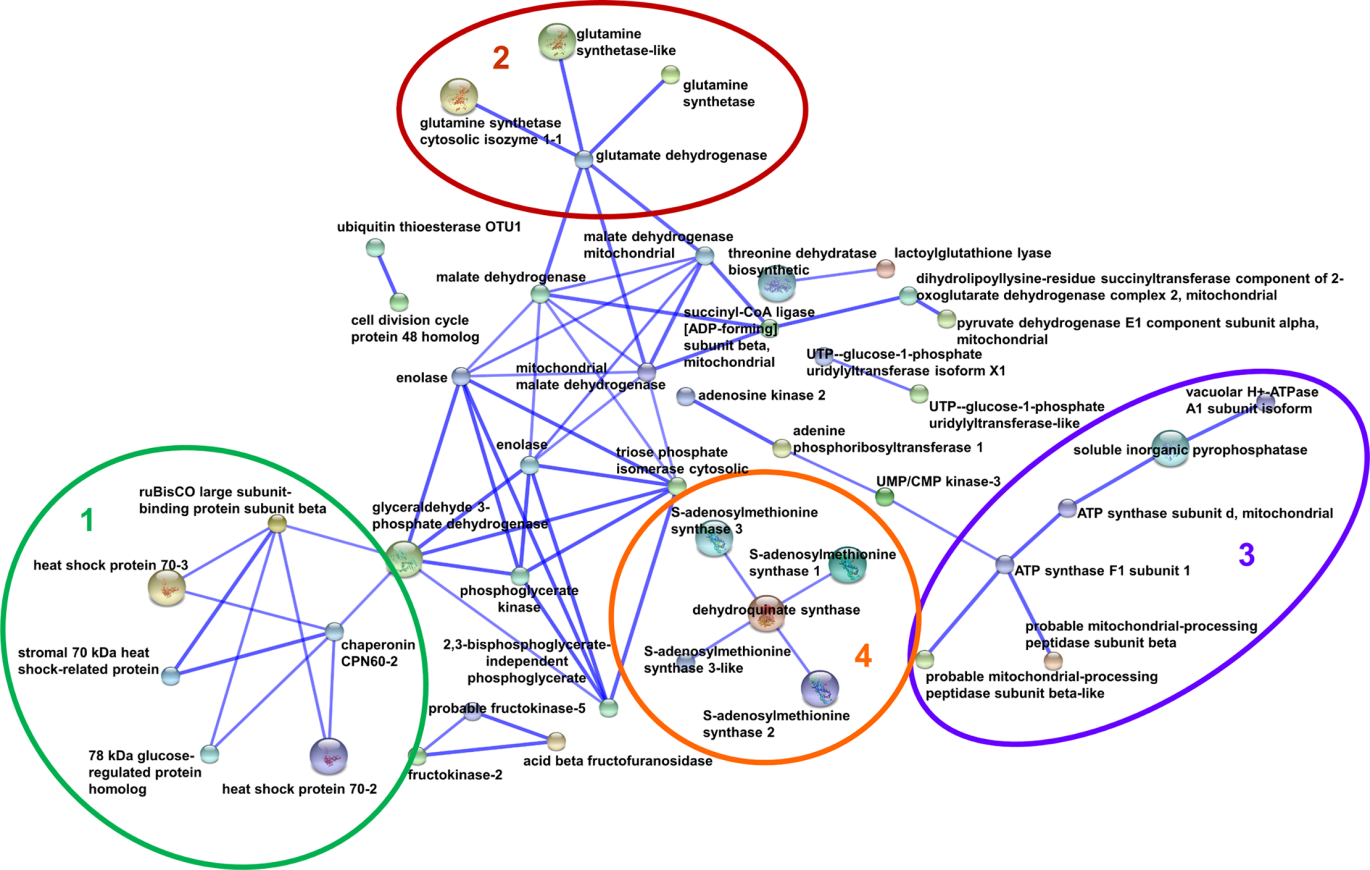
Interaction network of differentially represented proteins. The network was obtained using EMBL STRING with a confidence cut-off of 0.600.

Protein‑protein interactions were analyzed using the web resource STRING and 45 out of 96 identified proteins were connected in the interaction network (Fig 5). Notably, STRING analysis highlighted five main functional modules forming tightly connected clusters. The central module included proteins involved in the carbohydrate metabolism; the module 1 included proteins responsible for protein folding and degradation; the module 2 included proteins involved in nitrogen assimilation, the module 3 included proteins involved in S-adenosyl-L-methionine cycle/biosynthesis and, finally, the module 4 included proteins responsible for energy storage and production (Fig 5).

## Discussion

Growth and development of tomato plants is rather sensitive to the constant or transitory exposure to high temperatures that could lead to a drastic reduction of field production [1]. Temperatures exceeding 35°C negatively affects flower developmental processes and, anthers are the most susceptible reproductive organs [8,9,39]. Transcriptional analysis in tomato led to recognize that genes encoding heat stress transcription factors, heat shock proteins as well as proteins involved in ROS scavenger processes participate to the molecular mechanisms underlying response to HS and thermo-tolerance in reproductive tissues [10,11,15]. However, proteomic studies on HS response mechanisms in tomato reproductive tissues are still scarce [31] and just one paper by Zhou and colleagues reported on HS induced proteomic changes in tomato leaves [40]. In particular, the analysis of HS response in thermo-tolerant and thermo-sensitive plants has not been investigated yet.

In this light, we performed a differential proteomic study on anthers collected from flowers of thermo-tolerant (cv Saladette) and thermo-sensitive (cv M82) genotypes grown under CC and HT to unravel metabolic aspects and biological processes that allow tomato plants to face off with such adverse growth condition. Proteomic results highlighted that HT affected many metabolic pathways associated to energy production, nitrogen assimilation, glutamine and glutamate biosynthesis and cyanate degradation. In addition, HT modulated the expression of several proteins specialized in promoting refolding and proper protein assembly, thus preventing aggregation phenomena, and proteins involved in processes leading to ROS detoxification (Figs 2 and 5).

Moreover, the direct comparison of protein profiles of the two genotypes under CC and HT led to identify specific proteins that could be related to the tolerant features of SAL. In fact, thermo-tolerance traits could arise from the higher constitutively expression of proteins that can protect plants from the heat injury (basal thermo-tolerance) and/or the rapid and specific accumulation of proteins involved in stress response mechanisms (acquired thermo-tolerance) [10,11]. Therefore, proteins present in higher amount in SAL under CC and/or HT and whose expression was induced by HT could be regarded as potentially responsible of SAL thermo-tolerance. On the other hand, it cannot be ruled out that proteins exhibiting the opposite regulation, i.e. reduced expression under HT and higher level in M82 compared to SAL under CC and/or HT (such as caffeoyl-CoA O-methyltransferase 6 and polyphenol oxidase B and F), could also have a role in thermo-tolerance. In the present study, twenty-four proteins potentially related to the basal/acquired thermo-tolerance traits were identified (S4 Table) and most of them were involved in carbohydrate metabolism (enolase, glyceraldehyde 3-phosphate dehydrogenase), amino acid metabolism (glutamine and glutamate synthetases) and protein folding and degradation (chaperonin CPN60-2).

As to metabolic processes involved in abiotic stress response such as drought and high temperature, it is well documented that they determine alterations in anther sugar content and carbohydrate profile due to modifications in carbohydrate metabolism [7]. In our study, the abundance of many enzymes with key roles in glycolysis (glyceraldehyde 3-phosphate dehydrogenase, enolase, phosphoglycerate kinase, triose phosphate isomerase) and in TCA cycle (malate dehydrogenases) was modulated by HT. In addition, growth under HT altered the abundance of enzymes involved in sucrose degradation and galactose biosynthesis, such as fructokinases and fructofuranosidases (Table 1 and Fig 2). In plants, the content of soluble sugars is determinant to assure pollen development, viability and germination capacity. Imbalance in sugar metabolism caused by moderately elevated temperatures has been clearly associated with failure of tomato fruit set [9] and in particular, in the developing tomato anthers, the continuous exposure to high temperature has been related to an alteration in carbohydrate metabolism that contributed to the reduction of the number of pollen grains per flower and viability [41]. Changes in the amount of ATP synthase subunits also confirmed the importance of processes associated with energy production/storage (Fig 5).

S-adenosylmethionine synthetases (SAMSs) 1, 2 and 3 were overexpressed in both genotypes under HT and their amount was higher in M82 compared to SAL under HT. However, SAMS 2 and 3 were also constitutively more abundant in SAL and, therefore, a putative role in the thermo-tolerance of SAL could be suggested for these two proteins. In plants, these enzymes catalyse the conversion of L-methionine to S-adenosylmethionine that can be transformed through several biochemical reactions in polyamine, nicotianamine, ethylene, and, via the Yang cycle, in phytosiderophores. These compounds are reported to regulate plant tolerance to abiotic and biotic stresses [42–44]. Moreover, transgenic tomato plants overexpressing SAMS exhibited a significant increase in tolerance to alkali stress and maintained nutrient balance, higher photosynthetic capacity and lower oxidative stress compared with wild type plants [42]. Proteomic studies revealed that SAMSs were over-expressed in rice leaves under cold stress condition [45] and maize anthers after cold pre-treatment and subsequent cultivation [29].

Interestingly, in both genotypes under HT, we found an increase in the abundance of two glutamine synthetase isoforms and glutamate dehydrogenase, that were also constitutively present in higher amounts in the tolerant genotype. Glutamine synthetase and glutamate dehydrogenase catalyse the ammonia conversion from nitrate to glutamine and glutamate respectively, thus their function is strictly associated to nitrogen assimilation. In addition, another isoform of glutamine synthetase (Solyc05g1250.2) was significantly overexpressed in SAL compared to M82 under both growth conditions, although HT induced its expression only in M82. Our findings may suggest the pivotal position of nitrogen assimilation in HT response processes and the strict relation between these proteins, and in particular of glutamine synthetase isoform (Solyc05g1250.2), and the thermo-tolerance trait of SAL genotype (S4 Table).

Growth under HT leads to the production of ROS causing oxidative stress. Therefore, the response mechanisms to HT encompass the activation of biological systems for ROS detoxification. In this study, alterations in abundance of several enzymes involved in ascorbate and glutathione cycle (ascorbate peroxidases (APX) 1 and 2) and proteins with oxidoreductase activity (NAD(P)-linked oxidoreductase serfamily protein, peroxiredoxin-2E-2) in both genotypes confirmed a cross-talk between HS and oxidative stress signalling [11]. In fact, ROS could also play a key role in mediating important signal transduction events during abiotic stress aimed to activate stress-response pathways and induce defence mechanisms [46]. Through the ascorbate and glutathione cycle, which is one of the most important cellular mechanisms for hydrogen peroxide (H_2_O_2_) detoxification, ascorbate is oxidized to monodehydroascorbate by APX with the concomitant reduction of H_2_O_2_ to water. Increased transcriptional expression level of these enzymes has been reported in meiotic anthers of heat-tolerant and heat-sensitive tomato plants [10]. Moreover, an important role of ascorbate peroxidases in cold stress response has been observed also in rice and maize anthers [22,29].

The most direct consequence of HS is the misfolding of proteins that leads to the loss of protein functionality and the formation of protein aggregates. This causes an imbalance of protein homeostasis that is fundamental for cell development and survival. A complex network of molecular chaperones assures the proper protein de-novo folding or refolding and protein systems such as the ubiquitin–proteasome system assure degradation of irreversibly misfolded or aggregated proteins [47]. This proteomic study showed that as many as nineteen proteins belonged to the Folding, Sorting and Degradation functional category and most of them were overexpressed under HT, thus confirming their key role in the adaptation process. Among those, two 70kDa heat shock proteins, whose molecular function is the binding to unfolded proteins, and small heat shock proteins that act as co-chaperones, were overexpressed under HT, as also reported in other plant proteomic studies on stress response mechanisms [19,26,48].

Moreover, protein disulphide isomerase was more abundant after exposure to HT in both genotypes (Table 1). PDI acts as a direct folding catalyst in dithiol-disulfide interchange reactions promoting protein disulfide formation, isomerization or reduction. Its key role in conferring resistance to bacteria as well as to jasmonic acid and salicylic acid has been already reported in tomato cultivars [49].

Interestingly, among the proteins involved in protein folding and degradation identified in this study, mitochondrial chaperonin CPN60-2, serine protease inhibitor 5-like and multicystatin, whose chaperone function and protective role against environmental stresses have been previously reported [19], were overexpressed in SAL compared to M82 under both growth conditions and their expression was also triggered by HT in both genotypes, thus suggesting that they could significantly contribute to SAL thermo-tolerance features (S4 Table).

The reduced expression of polyphenol oxidase B and F under HT, observed in this study, could represent an additional aspect of plant response to adverse conditions. In fact, reduced activity of these enzymes inhibits the oxidation of phenols, whose accumulation has a protective role against HT damages in plant [2,50]. These enzymes were also present in lower amount in the tolerant genotype under CC and in similar amounts in both genotypes under HT, thus suggesting their contribution in the basal tolerance of SAL (S4 Table in Supporting Information). Similarly, caffeic acid 3-O-methyltransferase 5 and 6 also decreased their abundance in both genotypes grown under HT. These enzymes, involved in the synthesis of phenolic acids, are also responsible for the methylation of flavonoids, a critical step in the biosynthesis of lignin. Their down-regulation, due to abiotic or biotic stresses or to genetic modifications, has been associated to an alteration in the composition of lignin, caused by change in guaiacyl (G) and syringyl (S) monolignol subunits ratio that could have effects on total lignin content [51–53]. Therefore, our results suggested that HT could induce alterations in lignin biosynthesis, thus affecting cell wall thickness and rigidity.

In conclusion, our study highlights a deep cellular re-organization occurring in tomato anthers of both genotypes to face off with growth under HT, and contributes to identify proteins potentially involved in thermo-tolerance. Further proteomic studies performed on anthers of flowering plant such as tomato could widen the present knowledge on key genes and biochemical functions associated with thermo-tolerance, thus offering new perspectives for the generation of thermo-tolerant genotypes using breeding strategies or biotechnology approaches. Due to constantly increasing world warming, plants with enhanced tolerance features will surely have a prominent role in crop production in the next future.

## Supporting information

S1 Appendix Analysis of pollen viability of SAL and M82 genotypes grown under CC and HT. (PDF)

S2 Appendix Analysis of *Hsp17.6* transcript levels in anthers from SAL and M82 genotypes grown under CC and HT by qRT-PCR analysis. (PDF)

S1 Fig 2-DE gels of proteomes extracted from anthers of SAL and M82 genotypes. Two biological replicates and two technical replicates (16 2-DE gels) were run for each sample.
(PDF)

S1 Table Results of the 2-DE image analysis. Average spot volumes and standard deviation of the analyzed spots are reported.
(PDF)

S2 Table Identification of proteins differentially represented in SAL and M82 anther proteomes by PMF. Details of protein identification are reported.
(PDF)

S3 Table Identification of proteins differentially represented in SAL and M82 anther proteomes by nano-ESI-LC-MS/MS experiments. Details of protein identification are reported.
(PDF)

S4 Table Proteins involved in the thermo-tolerance features of SAL. Functional classification of the identified proteins and regulation of their abundance are reported.
(PDF)
