# Supplementary material for: Response mechanisms induced by exposure to high temperature in anthers from thermo-tolerant and thermo-sensitive tomato plants: A proteomic perspective
Source: PLoS One. 2018 Jul 19;13(7):e0201027. doi: 10.1371/journal.pone.0201027 (PMC6053223; doi:10.1371/journal.pone.0201027)

Analysis of pollen viability

Materials and Methods

Pollen viability was assessed on flowers of plants in CC/HT conditions of both genotypes by the p-Phenylenediamine method as described by Rodriguez-Riano and Dafni (Rodriguez-Riano T, Dafni A. A new procedure to assess pollen viability. Sex. Plant Reprod. 2000; 12:241–244). Briefly, pollen grains were released from anthers by mechanical shredding into staining buffer based on peroxidase indicator (Sigma 3901-10VL). The pollen suspension was incubated for 20 min at 37°C. Dark stained pollen was considered as viable. Viable and non-viable pollen grains were counted by a haemocytometer for a total count of no less than 200 pollen grains per flower for at least 4 flowers for each experimental condition/genotype. Staining percentage was determined by dividing the number of stained pollen grains by the total number of pollen grains per field of view and expressed as a percentage. Results are reported in Figure 1

Figure 1. Results of pollen viability test

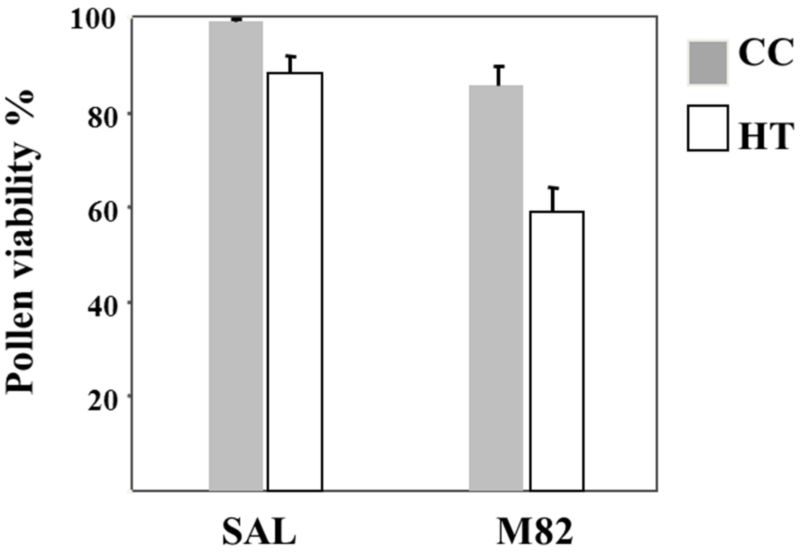

Supplement: S1 Appendix — (PDF) [file pone.0201027.s001.pdf]
