# Supplementary material for: Response mechanisms induced by exposure to high temperature in anthers from thermo-tolerant and thermo-sensitive tomato plants: A proteomic perspective
Source: PLoS One. 2018 Jul 19;13(7):e0201027. doi: 10.1371/journal.pone.0201027 (PMC6053223; doi:10.1371/journal.pone.0201027)

qRT-PCR analysis of Hsp17.6

Materials and Methods

Total RNA was extracted from 250 mg of anther tissue from CC and HT plants of both genotypes using RNeasy Plant Mini Kit (Qiagen, Hilden, Germany). RNA quantity was measured by NanoDrop ND-1000 Spectrophotometer (NanoDropTechnologies,Wilmington, DE, USA) and RNA integrity was verified on a denaturing MOPS/formaldehyde gel. The complementary DNA was synthesized from 1µg total RNA using QuantiTect Reverse Transcription Kit (Qiagen), according to manufacturer's instructions. For qRT-PCR, 4.5 µL of diluted (1:20) cDNA were used with 6.25 µL of 1X Platinum SYBR Green qPCR SuperMix (Life Technologies, Carlsbad, CA, USA) and 1.75 µL of primer mix (5µM) for each reaction. PCR was performed using ABI 7900 HT (Applied Biosystems). Two biological replicates with three technical replicates were tested. Quantification of gene expression was carried out using the $2^{-\Delta\Delta Ct}$ method (Livak KJ, Schmittgen TD. Analysis of relative gene expression data using real-time quantitative PCR and the 2(-Delta Delta C(T)). Methods. 2001, 25:402-408). *Lycopersicon esculentum* elongation Factor *EF1α* was used as endogenous reference gene for the normalization of the expression levels of the target genes. RNA extracted from plants grown in control condition (a) and Saladette (SAL) (b) served as calibrator sample for relative quantification of gene expression. Primers used are listed in Table 1 and results are reported in Figure 1.

Table 1. Primer sequences used for real-time quantitative PCR

**Gene name**	**Primer sequences**
Hsp17.6-C<br>(Solyc06g076560.1.1)	5'GATCAGCGGAGAGAGGAACG3'<br>5'- CCTCATGAATTTCCCGCTGC-3'
LeEF1<br>(X14449)	5'- TGATCAAGCCTGGTATGGTTGT -3'<br>5'- CTGGGTCATCCTTGGAGTT -3'

Figure 1. Changes in the relative expression levels of *Hsp 17.6* measured by qRT-PCR.
Variations are indicated in a) as fold-change in each genotype grown under HT with respect to CC condition; in b) as fold-change in M82 with respect to SAL under CC and HT. mRNA levels were calculated relatively to the expression of the Elongation factor *EF1α* used as reference gene.

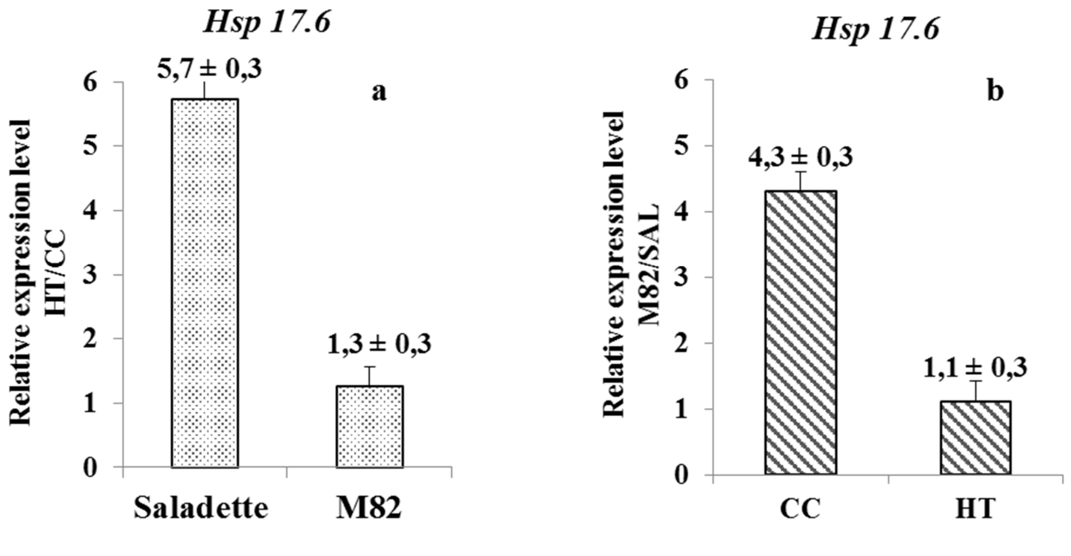

Supplement: S2 Appendix — (PDF) [file pone.0201027.s002.pdf]
