# Supplementary figures and images for: Response mechanisms induced by exposure to high temperature in anthers from thermo-tolerant and thermo-sensitive tomato plants: A proteomic perspective
Source: PLoS One. 2018 Jul 19;13(7):e0201027. doi: 10.1371/journal.pone.0201027 (PMC6053223; doi:10.1371/journal.pone.0201027)

A

M82\_CC

M82\_HT

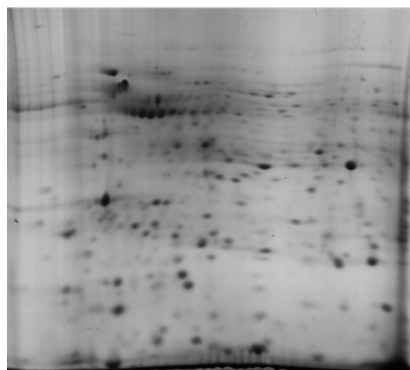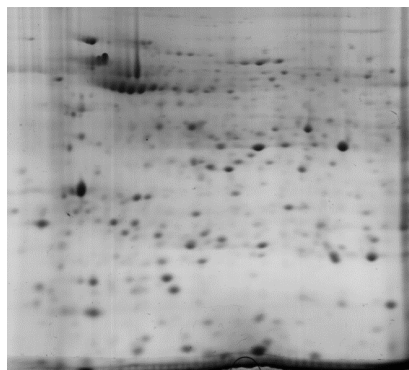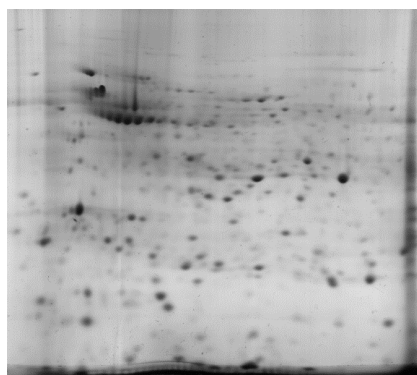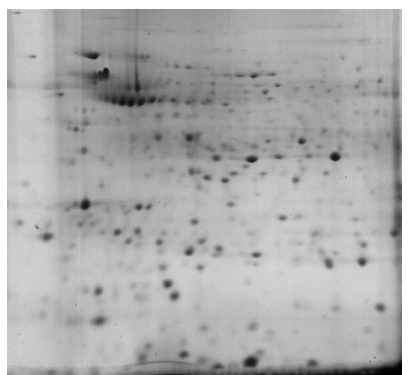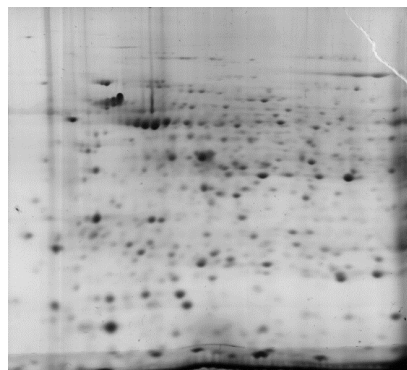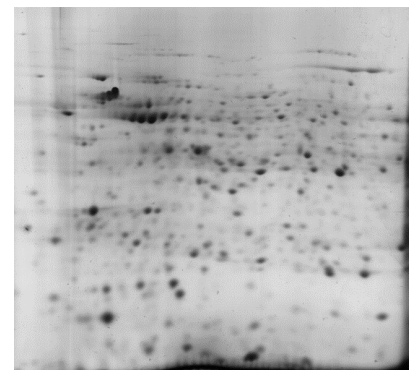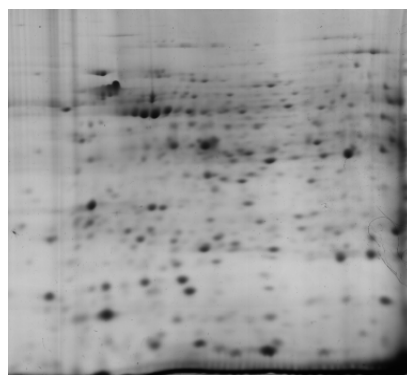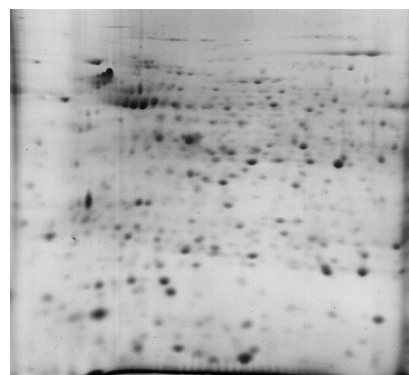

**B**

**SAL\_CC**

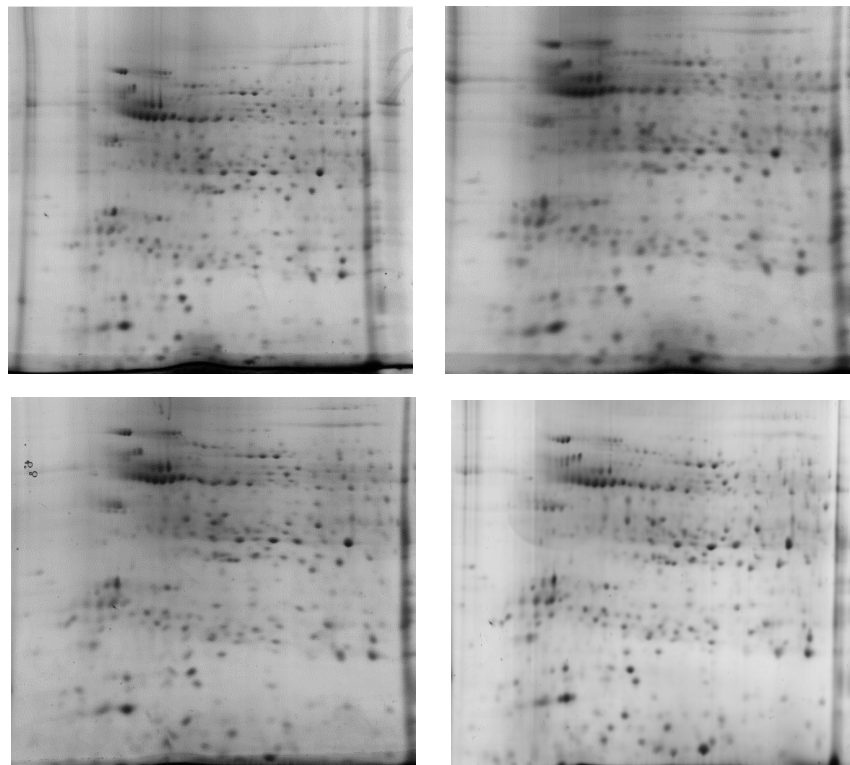

**SAL\_HT**

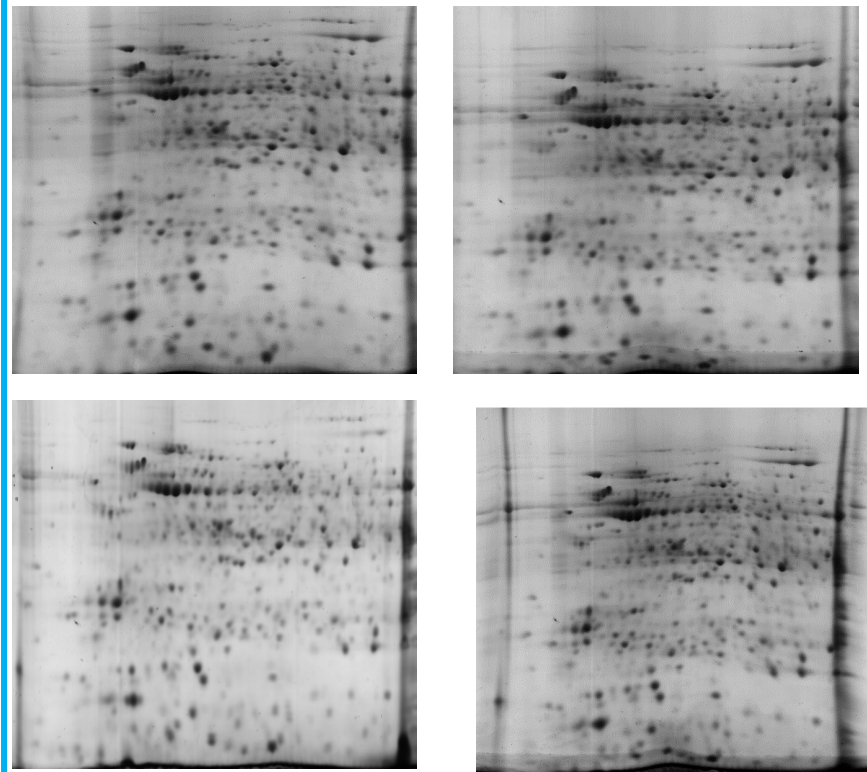

Supplement: S1 Fig — Two biological replicates and two technical replicates (16 2-DE gels) were run for each sample. (PDF) [file pone.0201027.s003.pdf]
