# Supplementary material for: Response mechanisms induced by exposure to high temperature in anthers from thermo-tolerant and thermo-sensitive tomato plants: A proteomic perspective
Source: PLoS One. 2018 Jul 19;13(7):e0201027. doi: 10.1371/journal.pone.0201027 (PMC6053223; doi:10.1371/journal.pone.0201027)
Supplement: S1 Table — Average spot volumes and standard deviation of the analyzed spots are reported. (PDF) [file pone.0201027.s004.pdf]

S1 Table. Results of the 2-DE Image analysis.
Average spot volumes and standard deviation of the analyzed spots are reported.

**M82_CC**	**M82 Control Condition**
**M82_HT**	**M82 High Temperature**
**SAL_CC**	**Saladette Control Condition**
**SAL_HT**	**Saladette High Temperature**

**spot number**	**sample**	**mean intensity**	**sd**
102	SAL_HT	18730543	1685453
102	SAL_CC	20729543	1865363
102	M_82_HT	11116120	1000155
102	M_82_CC	10115113	910063
103	SAL_HT	18647162	1677949
103	SAL_CC	38828667	3494284
103	M_82_HT	28643152	2577588
103	M_82_CC	29728767	2675292
201	SAL_HT	16902287	1520912
201	SAL_CC	4007161	360351
201	M_82_HT	9002287	809912
201	M_82_CC	5007161	450351
202	SAL_HT	45671396	4110130
202	SAL_CC	29715430	2674093
202	M_82_HT	72190195	6496822
202	M_82_CC	147543916	13278655
203	SAL_HT	14523339	1306807
203	SAL_CC	26634446	2396806
203	M_82_HT	15528339	1397257
203	M_82_CC	36634446	3296806
305	SAL_HT	12921479	1162638
305	SAL_CC	41006152	3690259
305	M_82_HT	27146309	2442873
305	M_82_CC	89597271	8063459
307	SAL_HT	64195741	5777324
307	SAL_CC	121215037	10909060
307	M_82_HT	33784741	3040334
307	M_82_CC	20894741	1880234
501	SAL_HT	8156833	733821
501	SAL_CC	4756748	427814
501	M_82_HT	15156833	1363821
501	M_82_CC	4246748	381914
702	SAL_HT	39148323	3523053
702	SAL_CC	8308887	747504
702	M_82_HT	76116899	6850225
702	M_82_CC	33932119	3053594
1004	SAL_HT	26493015	2384077
1004	SAL_CC	4007161	360351
1004	M_82_HT	24364761	2192535
1004	M_82_CC	4246748	381914
1202	SAL_HT	13840966	1245392

1202	SAL_CC	8046748	723912
1202	M_82_HT	19840966	1785392
1202	M_82_CC	4246748	381913
1203	SAL_HT	8205100	738163
1203	SAL_CC	4356848	391821
1203	M_82_HT	4246550	1368163
1203	M_82_CC	4246550	381911
1206	SAL_HT	10246387	921883
1206	SAL_CC	10847711	976002
1206	M_82_HT	10345377	930792
1206	M_82_CC	20847711	1875995
1301	SAL_HT	58061929	5225279
1301	SAL_CC	144944951	13044751
1301	M_82_HT	193505332	17415185
1301	M_82_CC	398667734	35879801
1401	SAL_HT	25468047	2291828
1401	SAL_CC	15457046	1390838
1401	M_82_HT	32123533	2890822
1401	M_82_CC	51222533	4609731
1501	SAL_HT	25226062	2270054
1501	SAL_CC	39131752	3521566
1501	M_82_HT	24326062	2189054
1501	M_82_CC	59132852	5321658
1504	SAL_HT	90361426	8132233
1504	SAL_CC	35245254	3171778
1504	M_82_HT	336725127	30304966
1504	M_82_CC	314497053	28304440
1702	SAL_HT	46335669	4169917
1702	SAL_CC	172881865	15559075
1702	M_82_HT	36917849	3322313
1702	M_82_CC	85591162	7702912
1703	SAL_HT	66374854	5973444
1703	SAL_CC	288456831	25960822
1703	M_82_HT	56374854	5073444
1703	M_82_CC	139534434	12557806
1704	SAL_HT	106613032	9594880
1704	SAL_CC	552809922	49752600
1704	M_82_HT	109212666	9828847
1704	M_82_CC	248648145	22378040
1707	SAL_HT	17584879	1582345
1707	SAL_CC	8662410	779323
1707	M_82_HT	18584879	1672345
1707	M_82_CC	9562410	860323
1901	SAL_HT	181382416	16324122
1901	SAL_CC	209981914	18898077
1901	M_82_HT	40691208	3661914
1901	M_82_CC	335928438	30233264
1902	SAL_HT	181482417	16333123
1902	SAL_CC	208981816	18808068
1902	M_82_HT	50560029	4550108

1902	M_82_CC	109981914	9898077
2101	SAL_HT	35919590	3232468
2101	SAL_CC	4007161	360350
2101	M_82_HT	73425068	6607961
2101	M_82_CC	20506467	1845287
2102	SAL_HT	12911967	1161784
2102	SAL_CC	34355488	3091701
2102	M_82_HT	12894978	1160255
2102	M_82_CC	11794978	1061255
2202	SAL_HT	32547102	2928947
2202	SAL_CC	58739277	5286243
2202	M_82_HT	33457002	3010838
2202	M_82_CC	68738267	6186145
2204	SAL_HT	24734336	2225797
2204	SAL_CC	22401545	2015846
2204	M_82_HT	52355103	4711666
2204	M_82_CC	46315244	4168079
2401	SAL_HT	13202513	1187934
2401	SAL_CC	4007161	360353
2401	M_82_HT	12201513	1097844
2401	M_82_CC	7007161	630346
2502	SAL_HT	6068136	545840
2502	SAL_CC	3246748	291916
2502	M_82_HT	11068136	995840
2502	M_82_CC	4246748	381909
2701	SAL_HT	102698686	9242587
2701	SAL_CC	56089131	5047727
2701	M_82_HT	36503835	3285050
2701	M_82_CC	120983257	10888198
2702	SAL_HT	172697686	15542497
2702	SAL_CC	96089131	8647727
2702	M_82_HT	79107549	7119384
2702	M_82_CC	209323770	18838844
2802	SAL_HT	27205945	2448241
2802	SAL_CC	4007161	360351
2802	M_82_HT	21943987	1974665
2802	M_82_CC	7801602	701850
2804	SAL_HT	9414763	847035
2804	SAL_CC	4845768	435825
2804	M_82_HT	18114763	1630035
2804	M_82_CC	4246748	381914
2806	SAL_HT	42091948	3787980
2806	SAL_CC	32001948	2879880
2806	M_82_HT	20639348	1857246
2806	M_82_CC	21538348	1938156
3102	SAL_HT	94080788	8466975
3102	SAL_CC	93080789	8376975
3102	M_82_HT	38027380	3422168
3102	M_82_CC	18389649	1654771
3201	SAL_HT	21084529	1897315

3201	SAL_CC	42378271	3813751
3201	M_82_HT	10468744	941894
3201	M_82_CC	13213273	1188902
3202	SAL_HT	22201472	1997840
3202	SAL_CC	42600697	3833770
3202	M_82_HT	6153737	553544
3202	M_82_CC	5955427	535696
3302	SAL_HT	18054020	1624569
3302	SAL_CC	29608137	2664439
3302	M_82_HT	4246748	381915
3302	M_82_CC	4186506	376493
3401	SAL_HT	21803831	1962053
3401	SAL_CC	4007161	360353
3401	M_82_HT	13852764	1246457
3401	M_82_CC	4246748	381909
3502	SAL_HT	17297859	1556515
3502	SAL_CC	9283604	835233
3502	M_82_HT	16297859	1466515
3502	M_82_CC	6286804	565514
3503	SAL_HT	25574500	2301410
3503	SAL_CC	27564500	2480510
3503	M_82_HT	17359426	1562053
3503	M_82_CC	13359426	1202053
3704	SAL_HT	14187603	1276590
3704	SAL_CC	6092638	548044
3704	M_82_HT	23048650	2074085
3704	M_82_CC	11399816	1025690
3801	SAL_HT	20771738	1869162
3801	SAL_CC	8220901	739587
3801	M_82_HT	13518469	1216368
3801	M_82_CC	4246748	381914
3803	SAL_HT	12547338	1128966
3803	SAL_CC	14143136	1272587
3803	M_82_HT	6273669	564335
3803	M_82_CC	13143136	1182587
3804	SAL_HT	7864582	707518
3804	SAL_CC	10864682	977527
3804	M_82_HT	18456111	1660755
3804	M_82_CC	9864582	887518
4004	SAL_HT	7154674	643625
4004	SAL_CC	9144684	822726
4004	M_82_HT	10716588	964197
4004	M_82_CC	20814578	1873015
4101	SAL_HT	22248264	2002052
4101	SAL_CC	38179154	3435832
4101	M_82_HT	22358264	2011952
4101	M_82_CC	48178164	4335736
4202	SAL_HT	20828119	1874239
4202	SAL_CC	34124263	3070892
4202	M_82_HT	21827119	1964149

4202	M_82_CC	54124263	4870885
4402	SAL_HT	48716230	4384166
4402	SAL_CC	47715230	4294076
4402	M_82_HT	149523477	13456818
4402	M_82_CC	89523477	8056818
4501	SAL_HT	20155288	1813683
4501	SAL_CC	9739139	876230
4501	M_82_HT	30099380	2708651
4501	M_82_CC	29099380	2618651
4504	SAL_HT	12868272	1157850
4504	SAL_CC	8246748	741912
4504	M_82_HT	30626487	2756089
4504	M_82_CC	4246748	381913
4602	SAL_HT	20090834	1807879
4602	SAL_CC	22190834	1996879
4602	M_82_HT	12195231	1097275
4602	M_82_CC	9194023	827183
4702	SAL_HT	20809370	1872549
4702	SAL_CC	35169798	3164988
4702	M_82_HT	21308390	1917461
4702	M_82_CC	45158598	4063980
5003	SAL_HT	31771719	2859160
5003	SAL_CC	4007161	360350
5003	M_82_HT	27676396	2490581
5003	M_82_CC	28676396	2580581
5104	SAL_HT	34787380	3130569
5104	SAL_CC	40202236	3617906
5104	M_82_HT	69656841	6268821
5104	M_82_CC	80650146	7258218
5201	SAL_HT	22821492	2053638
5201	SAL_CC	16881450	1519035
5201	M_82_HT	50032441	4502624
5201	M_82_CC	38850664	3496263
5202	SAL_HT	12286697	1105507
5202	SAL_CC	25839883	2325294
5202	M_82_HT	18622458	1675725
5202	M_82_CC	64638687	5817185
5203	SAL_HT	17382686	1564148
5203	SAL_CC	4007161	360351
5203	M_82_HT	33320435	2998545
5203	M_82_CC	4246748	381914
5303	SAL_HT	34938686	3144187
5303	SAL_CC	36463991	3281464
5303	M_82_HT	70467395	6341771
5303	M_82_CC	74965798	6746627
5401	SAL_HT	27018230	2431347
5401	SAL_CC	95620889	8605586
5401	M_82_HT	34430513	3098452
5401	M_82_CC	73334453	6599807
5403	SAL_HT	57155791	5143729

5403	SAL_CC	32638494	2937173
5403	M_82_HT	55155791	4963729
5403	M_82_CC	22628494	2036266
5405	SAL_HT	4600396	413743
5405	SAL_CC	11899232	1070638
5405	M_82_HT	6700386	602742
5405	M_82_CC	12899232	1160638
5502	SAL_HT	14230485	1280452
5502	SAL_CC	4007161	360353
5502	M_82_HT	27692976	2492076
5502	M_82_CC	4246748	381909
5704	SAL_HT	4007161	360349
5704	SAL_CC	3479996	312904
5704	M_82_HT	9799012	881615
5704	M_82_CC	11816257	1063166
5801	SAL_HT	14179819	1275891
5801	SAL_CC	47058550	4234977
5801	M_82_HT	10895029	980260
5801	M_82_CC	23049910	2074199
6203	SAL_HT	23310839	2097681
6203	SAL_CC	27142819	2442559
6203	M_82_HT	7261045	653199
6203	M_82_CC	11052490	994429
6301	SAL_HT	30279829	2724892
6301	SAL_CC	28279830	2544892
6301	M_82_HT	14237924	1281120
6301	M_82_CC	15137852	1362114
6303	SAL_HT	17974092	1617375
6303	SAL_CC	38290105	3445816
6303	M_82_HT	3577528	321685
6303	M_82_CC	9092063	817993
6403	SAL_HT	30802449	2771925
6403	SAL_CC	26610585	2394658
6403	M_82_HT	3591737	322962
6403	M_82_CC	1957590	175889
6405	SAL_HT	48533574	4367729
6405	SAL_CC	165414521	14887014
6405	M_82_HT	38533574	3467729
6405	M_82_CC	82414521	7417014
6504	SAL_HT	5586506	502493
6504	SAL_CC	9586506	862493
6504	M_82_HT	12357872	1111916
6504	M_82_CC	18357872	1651916
6604	SAL_HT	70116602	6310201
6604	SAL_CC	29409060	2646522
6604	M_82_HT	30519907	2746499
6604	M_82_CC	12508251	1125450
6701	SAL_HT	30524734	2746930
6701	SAL_CC	63001089	5669802
6701	M_82_HT	14835715	1334918

6701	M_82_CC	31177529	2805681
6703	SAL_HT	30674354	2760396
6703	SAL_CC	64101079	5768801
6703	M_82_HT	12524734	1126930
6703	M_82_CC	32001089	2879801
6705	SAL_HT	15935715	1433918
6705	SAL_CC	28277529	2544682
6705	M_82_HT	14835715	1334918
6705	M_82_CC	31177529	2805681
6706	SAL_HT	6314921	568051
6706	SAL_CC	19866177	1787664
6706	M_82_HT	4246748	381916
6706	M_82_CC	16774268	1509385
6805	SAL_HT	11656350	1048779
6805	SAL_CC	10646350	957879
6805	M_82_HT	11991877	1078976
6805	M_82_CC	38643232	3477598
7002	SAL_HT	16992065	1528991
7002	SAL_CC	4007161	360350
7002	M_82_HT	8360050	752110
7002	M_82_CC	7060050	635110
7201	SAL_HT	19465044	1751562
7201	SAL_CC	4007161	360353
7201	M_82_HT	27604041	2484072
7201	M_82_CC	4246748	381909
7203	SAL_HT	15656726	1408813
7203	SAL_CC	4007161	360353
7203	M_82_HT	15739650	1416277
7203	M_82_CC	4246748	381909
7301	SAL_HT	30807380	2772372
7301	SAL_CC	51990145	4678821
7301	M_82_HT	30909280	2781543
7301	M_82_CC	61990122	5578812
7403	SAL_HT	59943982	5394666
7403	SAL_CC	35133771	3161747
7403	M_82_HT	69933882	6293757
7403	M_82_CC	34132871	3071659
7404	SAL_HT	6444686	579730
7404	SAL_CC	13193761	1187147
7404	M_82_HT	4648134	418040
7404	M_82_CC	13290208	1195820
7502	SAL_HT	13604055	1224070
7502	SAL_CC	7507055	675340
7502	M_82_HT	23329839	2099391
7502	M_82_CC	4246748	381913
7503	SAL_HT	15123850	1360854
7503	SAL_CC	7946748	714914
7503	M_82_HT	19127350	1721169
7503	M_82_CC	4246748	381909
7601	SAL_HT	17363037	1562381

7601	SAL_CC	8961252	806221
7601	M_82_HT	31363037	2822381
7601	M_82_CC	7561252	680214
7701	SAL_HT	58061929	5225280
7701	SAL_CC	48061929	4325280
7701	M_82_HT	15205100	1368165
7701	M_82_CC	28205100	2538165
7704	SAL_HT	11593788	1043149
7704	SAL_CC	6243579	561630
7704	M_82_HT	14593788	1313149
7704	M_82_CC	3243579	291624
8105	SAL_HT	83383954	7504261
8105	SAL_CC	51561382	4640229
8105	M_82_HT	40461282	3641220
8105	M_82_CC	73483854	6613252
8201	SAL_HT	13684458	1231307
8201	SAL_CC	8146748	732913
8201	M_82_HT	23684458	2131307
8201	M_82_CC	4246748	381914
8203	SAL_HT	40451282	3640319
8203	SAL_CC	47451383	4270329
8203	M_82_HT	86373954	7773360
8203	M_82_CC	76363854	6872450
8301	SAL_HT	13463401	1211706
8301	SAL_CC	32182190	2896397
8301	M_82_HT	6657861	599207
8301	M_82_CC	8325889	749330
8402	SAL_HT	18656191	1678764
8402	SAL_CC	4007161	360352
8402	M_82_HT	6817283	613263
8402	M_82_CC	3907161	351352
8403	SAL_HT	14363502	1292422
8403	SAL_CC	33281150	2995011
8403	M_82_HT	7048963	634114
8403	M_82_CC	8445879	759836
8404	SAL_HT	42163247	3794396
8404	SAL_CC	28105618	2529210
8404	M_82_HT	52163247	4694396
8404	M_82_CC	18105618	1629209
8501	SAL_HT	46512193	4185804
8501	SAL_CC	47512193	4275804
8501	M_82_HT	23992257	2159010
8501	M_82_CC	22322257	2008710
8601	SAL_HT	12442565	1119538
8601	SAL_CC	27803137	2501989
8601	M_82_HT	21410945	1926692
8601	M_82_CC	10309984	927606
8701	SAL_HT	10438547	939177
8701	SAL_CC	27224832	2449943
8701	M_82_HT	11437537	1029086

8701	M_82_CC	17224832	1549936
8703	SAL_HT	9133133	821688
8703	SAL_CC	14185997	1276446
8703	M_82_HT	9223133	829788
8703	M_82_CC	24186897	2176527
8801	SAL_HT	15961279	1436220
8801	SAL_CC	3935986	353944
8801	M_82_HT	10935904	983937
8801	M_82_CC	9718056	874330
8901	SAL_HT	83913047	7551878
8901	SAL_CC	30490457	2743845
8901	M_82_HT	38086152	3427458
8901	M_82_CC	18950044	1705207
8902	SAL_HT	93973374	8457308
8902	SAL_CC	30490457	2743845
8902	M_82_HT	46662930	4199368
8902	M_82_CC	20950044	1885207
8903	SAL_HT	83973374	7557308
8903	SAL_CC	20490457	1843845
8903	M_82_HT	36662930	3299368
8903	M_82_CC	10950044	985207
9501	SAL_HT	33342764	3000557
9501	SAL_CC	20590696	1852871
9501	M_82_HT	43342764	3900557
9501	M_82_CC	11590696	1042864
9601	SAL_HT	26035497	2342900
9601	SAL_CC	46035497	4142900
9601	M_82_HT	16035497	1442900
9601	M_82_CC	12243863	1101653
9702	SAL_HT	43354425	3901604
9702	SAL_CC	22687451	2041577
9702	M_82_HT	73464525	6611513
9702	M_82_CC	22878551	2058776

spot number	SAL_HT average intensity*	sd	SAL_CC average intensity*	sd	M_82_HT average intensity*	sd	M_82_CC average intensity*	sd
1702								
1703	219323555	30697558	1014148618	194758593	202505369	37409780	473773741	83069507
1704								
2701	275396372	49496768	152178262	28284271	115611384	30125375	330307027	62466176
2702								
1901	362864833	70711	418963730	707176	91251237	6978310	445910352	159768319
1902								
305	70983408	31919118	185951103	73495830	220651641	117633593	488265005	218545820
1301								
6701	61199088	105797	127102168	777810	27360449	1634110	63178618	582345
6703								
8301	27826903	636468	65463340	777082	13706824	276551	16771768	84846
8403								
8901	261859795	5790996	81471371	5773503	121412012	5409662	50856782	5293187
8902								
8903								

* this is the sum of average mean intensities of these spots that contained the same proteins
