# Supplementary material for: Response mechanisms induced by exposure to high temperature in anthers from thermo-tolerant and thermo-sensitive tomato plants: A proteomic perspective
Source: PLoS One. 2018 Jul 19;13(7):e0201027. doi: 10.1371/journal.pone.0201027 (PMC6053223; doi:10.1371/journal.pone.0201027)
Supplement: S2 Table — Details of protein identification are reported. (PDF) [file pone.0201027.s005.pdf]

Table S2: Identification by PMF of proteins differentially expressed in Saladette and M82 tomato anthers grown under high temperature and control conditions

Spot	Accession number (NCBInr)	Protein	Theoretical Mr (Da)	Theoretical pI	Score	Matched peptides / searched m/z values	Sequence Coverage (%)	Source
102	XP_015069432.1	pollen allergen Che a 1-like	20315	4.65	105	6/13	49	*Solanum pennellii*
103	XP_004239074.1	nodulin-related protein 1	15879	4.57	114	8/40	69	*Solanum lycopersicum*
201	XP_004247428.1	uncharacterized protein OsI_027940-like	23950	4.27	101	8/40	44	*Solanum lycopersicum*
202	XP_004249331.1	nascent polypeptide-associated complex subunit alpha-like protein-like	21749	4.39	108	8/34	54	*Solanum lycopersicum*
203	XP_004244803.1	proteasome subunit alpha type-5-like	26223	4.71	142	10/29	56	*Solanum lycopersicum*
305	NP_001234183.1	plastid lipid associated protein CHRC	35685	5.26	105	11/27	32	*Solanum lycopersicum*
307	XP_004251245.1	elongation factor 1-beta 2	24562	4.63	112	7/14	34	*Solanum lycopersicum*
501	XP_004230497.1	ankyrin repeat domain-containing protein 2-like	37353	4.43	115	8/15	25	*Solanum lycopersicum*
702	XP_004232206.1	uncharacterized protein At5g39570	41775	4.66	180	15/40	37	*Solanum lycopersicum*
1004	XP_010312248.1	peroxiredoxin-2E-2, chloroplastic	24649	8.60	121	8/20	44	*Solanum lycopersicum*
1202	NP_001266269.1	inducible plastid-lipid associated protein	18301	5.81	100	8/31	43	*Solanum lycopersicum*
1203	XP_004244120.1	ubiquitin thioesterase OTU1	23552	5.06	129	8/16	40	*Solanum lycopersicum*
1206	XP_004230104.1	probable ribose-5-phosphate isomerase 3, chloroplastic	31185	6.00	119	8/17	32	*Solanum lycopersicum*
1301	NP_001234183.1	plastid lipid associated protein CHRC	35685	5.26	149	12/31	40	*Solanum lycopersicum*

1401	NP_001316365.1	dehydrin	23098	5.13	218	13/31	66	*Solanum lycopersicum*
1501	NP_001316163.1	N2-acetylornithine deacetylase	51082	5.00	108	14/60	28	*Solanum lycopersicum*
1504	NP_001269248.1	abscicic acid stress ripening protein 4	32981	4.95	149	14/40	56	*Solanum lycopersicum*
1702	NP_001233862.2	leucine aminopeptidase 1, chloroplastic	60542	5.71	226	20/55	46	*Solanum lycopersicum*
1703	NP_001233862.2	leucine aminopeptidase 1, chloroplastic	60542	5.71	224	20/53	44	*Solanum lycopersicum*
1704	NP_001233862.2	leucine aminopeptidase 1, chloroplastic	60542	5.71	201	16/60	47	*Solanum lycopersicum*
1707	XP_004240392.1	protein disulfide-isomerase-like	55108	4.81	273	25/60	43	*Solanum lycopersicum*
1901	XP_004230445.1	stromal 70 kDa heat shock-related protein, chloroplastic	74965	5.20	153	17/44	22	*Solanum lycopersicum*
1902	XP_004230445.1	stromal 70 kDa heat shock-related protein, chloroplastic	74965	5.20	163	19/47	26	*Solanum lycopersicum*
2101	NP_001233872.1	mitochondrial small heat shock protein	23818	6.47	123	9/26	50	*Solanum lycopersicum*
2102	XP_004251263.1	ATP synthase subunit d, mitochondrial-like	19781	5.33	118	11/32	52	*Solanum lycopersicum*
2202	XP_004248757.1	caffeoyl-CoA O-methyltransferase 5	27929	5.14	113	11/39	40	*Solanum lycopersicum*
2204	NP_001296294.1	Oxygen-evolving enhancer protein 1, chloroplastic	35154	5.91	176	14/32	43	*Solanum lycopersicum*
2401	XP_004249586.1	adenosine kinase 2	37962	5.07	173	14/50	61	*Solanum lycopersicum*
2502	XP_019068856.1	fumarylacetoacetase-like	46553	5.07	105	9/25	20	*Solanum lycopersicum*
2701	NP_001296095.1	Threonine dehydratase biosynthetic, chloroplastic	65181	5.25	210	19/45	35	*Solanum lycopersicum*
2702	NP_001296095.1	threonine dehydratase biosynthetic, chloroplastic	65181	5.25	166	18/55	30	*Solanum lycopersicum*
2802	XP_004250958.1	heat shock cognate 70 kDa protein 2-like	71498	5.10	124	13/28	28	*Solanum lycopersicum*

2804	NP_001266213.1	heat shock protein 70-3	71744	5.14	102	15/27	22	*Solanum lycopersicum*
2806	P49118.1	78 kDa glucose-regulated protein homolog	73475	5.10	118	14/34	21	*Solanum lycopersicum*
3102	XP_004235415.1	serine protease inhibitor 5-like	23117	5.10	103	6/12	41	*Solanum lycopersicum*
3201	XP_004230885.1	triose phosphate isomerase chloroplastic	35043	6.45	101	11/60	34	*Solanum lycopersicum*
3202	XP_004252616.1	soluble inorganic pyrophosphatase PPA1	24520	5.30	109	8/16	34	*Solanum lycopersicum*
3302	XP_004232138.1	alpha-1,4-glucan-protein synthase [UDP-forming] 1-like	40824	5.38	195	14/27	33	*Solanum lycopersicum*
3401	NP_001234293.2	succinyl-CoA ligase [ADP-forming] subunit beta, mitochondrial	45597	6.09	101	10/30	27	*Solanum lycopersicum*
3502	NP_001310599.1	glutamine synthetase	47852	6.29	93	9/31	20	*Solanum lycopersicum*
3503	NP_001317048.1	actin-41	41858	5.31	152	13/37	37	*Solanum lycopersicum*
3704	XP_004228946.1	ruBisCO large subunit-binding protein subunit beta, chloroplastic	63238	5.72	198	19/29	37	*Solanum lycopersicum*
3801	XP_004247810.1	chaperonin CPN60-2, mitochondrial	61807	5.51	181	19/38	34	*Solanum lycopersicum*
3803	NP_001234281.2	vacuolar H+-ATPase A1 subunit isoform;	68798	5.20	164	18/36	33	*Solanum lycopersicum*
3804	NP_001315608.1	cell division cycle protein 48 homolog	90147	5.10	136	14/26	19	*Solanum lycopersicum*
4004	AFJ93093.1	proteinase inhibitor II	17209	7.33	106	7/23	56	*Solanum lycopersicum*
4101	NP_001316139.1	adenine phosphoribosyltransferase 1	19713	5.33	163	12/41	66	*Solanum lycopersicum*
4202	XP_004230766.1	caffeoyl-CoA O-methyltransferase 6	28116	5.43	169	13/48	62	*Solanum lycopersicum*
4402	XP_004241231.1	actin-82	41959	5.29	111	11/33	25	*Solanum lycopersicum*
4501	NP_001234843.2	acid beta-fructofuranosidase precursor	70338	5.54	143	16/42	28	*Solanum lycopersicum*

4504	NP_001296305.1	adenosylmethionine synthase 2	43511	5.41	179	14/34	41	*Solanum lycopersicum*
4602	XP_004237119.2	Web family protein At1g12150-like	71821	5.29	132	13/24	22	*Solanum lycopersicum*
4702	Mixture<br>XP_004251648.1<br>XP_006468800.1	mitochondrial-processing peptidase subunit alpha<br>ATP synthase subunit beta, mitochondrial-like	54869<br>59928	6.05<br>6.06	112<br>105	12/42<br>11/42	26<br>26	*Solanum lycopersicum*<br>*Citrus sinensi*
5003	NP_001234225.1	type I small heat shock protein 17.6 kDa isoform	17634	5.84	140	13/43	59	*Solanum lycopersicum*
5104	XP_004251703.1	20 kDa chaperonin, chloroplastic	26626	8.55	156	12/40	57	*Solanum lycopersicum*
5201	NP_001234231.1	remorin 1	21837	5.64	161	13/28	62	*Solanum lycopersicum*
5202	XP_010324012.1	stress-response A/B barrel domain-containing protein UP3-like	29196	7.10	158	12/40	42	*Solanum lycopersicum*
5203	NP_001296993.1	carbonic anhydrase, 2 1	29904	5.63	283	22/30	74	*Solanum lycopersicum*
5303	NP_001234104.1	annexin p34	35894	5.39	229	19/39	55	*Solanum lycopersicum*
5401	NP_001317632.1	NAD(P)-linked oxidoreductase superfamily protein	40909	7.57	146	13/36	35	*Solanum lycopersicum*
5403	NP_001309987.1	glutamine synthetase cytosolic isozyme 1-1	39471	5.62	107	10/24	29	*Solanum lycopersicum*
5405	NP_001316520.1	phosphoglycerate kinase	42263	5.78	167	11/22	44	*Solanum lycopersicum*
5502	NP_001234425.1	adenosylmethionine synthase 1	43730	5.52	119	13/34	35	*Solanum lycopersicum*
5704	XP_004243405.2	seed biotin-containing protein SBP65-like	56381	5.43	185	18/40	38	*Solanum lycopersicum*
5801	XP_004243619.1	2-3bisphosphoglycerate-independent phosphoglycerate mutase	61275	5.59	174	17/38	29	*Solanum lycopersicum*
6203	NP_001296740.1	UMP/CMP kinase 3	22972	5.76	115	9/40	54	*Solanum lycopersicum*
6301	NP_001233888.1	fructokinase-2	34969	5.79	284	20/39	70	*Solanum lycopersicum*

6303	XP_010313061.1	probable fructokinase-5	35180	6.16	290	24/60	68	*Solanum lycopersicum*
6403	XP_004240034.1	glutamine synthetase-like	39213	5.79	123	11/40	30	*Solanum lycopersicum*
6405	NP_001234001.2	mitochondrial malate dehydrogenase	36287	8.73	156	13/44	50	*Solanum lycopersicum*
6504	NP_001332774.1	enolase	48206	5.99	106	14/30	32	*Solanum lycopersicum*
6604	NP_001234080.1	enolase	48054	5.68	174	16/36	50	*Solanum lycopersicum*
6701	XP_015064515.1	probable mitochondrial-processing peptidase subunit beta	59202	6.05	131	14/27	23	*Solanum pennellii*
6703	XP_015064515.1	probable mitochondrial-processing peptidase subunit beta	59202	6.05	156	18/46	32	*Solanum pennellii*
6705	XP_004239065.1	probable mitochondrial-processing peptidase subunit beta-like	59716	6.16	204	20/50	35	*Solanum lycopersicum*
6706	YP_009430460.1	ATP synthase F1 subunit 1	55699	5.93	122	14/45	31	*Solanum lycopersicum*
6805	NP_001318059.1	polyphenol oxidase F, chloroplastic	66950	6.04	186	20/42	36	*Solanum lycopersicum*
7002	NP_001234503.1	eukaryotic translation initiation factor 5A-2	17714	5.78	106	7/19	45	*Solanum lycopersicum*
7201	NP_001234782.1	cytosolic ascorbate peroxidase 1	27733	5.61	144	15/31	58	*Solanum lycopersicum*
7203	NP_001234788.2	cytosolic ascorbate peroxidase 2	27532	6.00	108	10/35	38	*Solanum lycopersicum*
7301	XP_004232705.1	putative lactoylglutathione lyase-like	32953	5.95	156	16/60	55	*Solanum lycopersicum*
7403	XP_004247734.1	malate dehydrogenase	35703	5.91	112	10/29	33	*Solanum lycopersicum*
7404	NP_001233863.1	3-dehydroquinate synthase, chloroplastic	48492	8.35	100	11/37	23	*Solanum lycopersicum*
7502	NP_001234004.1	S-adenosylmethionine synthetase 3	43082	5.76	121	11/41	37	*Solanum lycopersicum*
7503	XP_004244101.1	dihydrolipoyllysine-residue succinyltransferase component of 2-	51387	9.08	107	12/40	22	*Solanum lycopersicum*

		oxoglutarate dehydrogenase complex 2, mitochondrial-like						
7601	XP_004250240.1	UTP--glucose-1-phosphate uridylyltransferase isoform X1	52014	5.384	246	19/29	42	*Solanum lycopersicum*
7701	NP_001234691.2	wound-inducible carboxypeptidase	56039	5.84	106	8/18	22	*Solanum lycopersicum*
7704	NP_001233884.2	Leucine aminopeptidase 2, chloroplastic;	60082	8.18	132	13/25	28	*Solanum lycopersicum*
8105	XP_004234310.1	gamma carbonic anhydrase-like 2, mitochondrial	27864	8.54	103	9/40	34	*Solanum lycopersicum*
8201	NP_001233847.1	carbonic anhydrase	28908	6.01	128	11/26	43	*Solanum lycopersicum*
8203	XP_004232424.1	gamma carbonic anhydrase 1, mitochondrial-like	29415	6.25	117	7/11	31	*Solanum lycopersicum*
8301	NP_001315977.1	malate dehydrogenase, mitochondrial	35860	8.90	130	12/31	41	*Solanum lycopersicum*
8402	NP_001296789.1	pyruvate dehydrogenase E1 component subunit alpha, mitochondrial	43745	7.16	137	16/46	32	*Solanum lycopersicum*
8403	NP_001315977.1	malate dehydrogenase, mitochondrial	35860	8.90	139	9/15	31	*Solanum lycopersicum*
8404	NP_001266254.2	glyceraldehyde 3-phosphate dehydrogenase	36822	6.34	102	10/30	38	*Solanum lycopersicum*
8501	XP_004239981.3	pectinesterase 1-like	40660	6.26	128	10/23	28	*Solanum lycopersicum*
8601	XP_004239832.1	UTP--glucose-1-phosphate uridylyltransferase-like	52328	5.83	134	13/40	30	*Solanum lycopersicum*
8701	NP_001296718.1	beta-D-xylosidase 2 precursor	86179	8.45	114	12/40	23	*Solanum lycopersicum*
8703	NP_001296326.1	Polyphenol oxidase B, chloroplastic;	67812	6.92	120	13/39	28	*solanum Lycopersicon*
8801	XP_004245731.1	hsp70-Hsp90 organizing protein 2	65410	5.99	260	27/60	54	*Solanum lycopersicum*
8901	XP_004253396.2	multicystatin, partial	84879	5.76	188	19//29	29	*Solanum lycopersicum*
8902	XP_004253396.2	multicystatin, partial	84879	5.76	233	23/34	39	*Solanum lycopersicum*

8903	XP_004253396.2	multicystatin, partial	84879	5.76	220	22/33	35	*Solanum lycopersicum*
9501	NP_001292722.1	glutamate dehydrogenase	44878	6.20	122	14/30	34	*Solanum lycopersicum*
9601	XP_010312254.1	S-adenosylmethionine synthase 3-like	43089	6.12	128	11/40	44	*Solanum lycopersicum*
9702	NP_001333533.1	aldehyde dehydrogenase family 2 member B7d	58204	8.0	168	15/35	32	*Solanum lycopersicum*
