# Supplementary material for: Response mechanisms induced by exposure to high temperature in anthers from thermo-tolerant and thermo-sensitive tomato plants: A proteomic perspective
Source: PLoS One. 2018 Jul 19;13(7):e0201027. doi: 10.1371/journal.pone.0201027 (PMC6053223; doi:10.1371/journal.pone.0201027)
Supplement: S3 Table — Details of protein identification are reported. (PDF) [file pone.0201027.s006.pdf]

Table S3: Identification by nano-ESI-LC-MS/MS of proteins differentially expressed in Saladette and M82 tomato anthers grown under high temperature and control conditions

**Spot**	**Accession number**	**Protein**	**Theoretical Mr (Da)**	**Theoretical pI**	**Score**	**Sequence Coverage (%)**	**Matched peptides**	**Source**
202	XP_004249331.1	nascent polypeptide-associated complex subunit alpha-like protein-like	21749	4.39	157	21	2 matches<br>2 sequences	*Solanum Lycopersicum*
305	NP_001234183.1	plastid lipid associated protein CHRC	35685	5.26	139	8	5 matches<br>2 sequences	*Solanum Lycopersicum*
702	XP_004232206.1	uncharacterized protein At5g39570	41775	4.66	66	4	1 matches<br>1sequences	*Solanum Lycopersicum*
1401	NP_001316365.1	dehydrin	23098	5.13	206	17	3 matches<br>3sequences	*Solanum Lycopersicum*
1901	XP_015054863.1	stromal 70 kDa heat shock-related protein, chloroplastic	74966	5.16	100	2	16 matches<br>1 sequences	*Solanum pennellii*
2202	XP_004248757.1	caffeoyl-CoA O-methyltransferase 5	27929	5.14	64	3	1 matches<br>1 sequences	*Solanum Lycopersicum*
2401	XP_004249586.1	adenosine kinase 2	37962	5.07	96	5	1 matches<br>1 sequences	*Solanum Lycopersicum*
3102	XP_004235415.1	serine protease inhibitor 5-like	23117	5.10	422	23	3 matches<br>3 sequences	*Solanum Lycopersicum*
3202	XP_004252616.1	soluble inorganic pyrophosphatase PPA1-like isoform X1	23795	5.60	137	11	2 matches<br>2 sequences	*Solanum Lycopersicum*
3201	XP_004236746.1	triose phosphate isomerase cytosolic	35043	6.45	121	5	1 matches<br>1sequences	*Solanum Lycopersicum*
3704	XP_004251160.1	ruBisCO large subunit-binding protein subunit alpha	62032	5.21	177	6	4 matches<br>3 sequences	*Solanum Lycopersicum*
3801	XP_004247810.1	chaperonin CPN60-2, mitochondrial	61807	5.51	211	5	3 matches<br>3 sequences	*Solanum Lycopersicum*

3804	NP_001315608.1	cell division cycle protein 48 homolog	90147	5.10	206	2	2 matches<br>2 sequences	*Solanum Lycopersicum*
4202	XP_004230766.1	caffeoyl-CoA O-methyltransferase 6	28116	5.43	99	6	3 matches<br>1 sequences	*Solanum Lycopersicum*
5104	XP_004251703.1	20 kDa chaperonin, chloroplastic	26626	8.55	54	4	1 matches<br>1 sequences	*Solanum Lycopersicum*
5202	XP_010324012.1	stress-response A/B barrel domain-containing protein UP3-like	29196	7.10	62	4	1 matches<br>1 sequences	*Solanum Lycopersicum*
5401	NP_001317632.1	NAD(P)-linked oxidoreductase superfamily protein	40909	7.57	815	21	7 matches<br>7 sequences	*Solanum Lycopersicum*
5403	NP_001309987.1	glutamine synthetase cytosolic isozyme 1-1	39471	5.62	190	8	4 matches<br>3 sequences	*Solanum Lycopersicum*
6303	XP_010313061.1	probable fructokinase-5	35180	6.16	123	8	2 matches<br>2 sequences	*Solanum Lycopersicum*
6403	XP_004240034.1	glutamine synthetase-like	39213	5.79	68	7	1 matches<br>1 sequences	*Solanum Lycopersicum*
6405	NP_001234001.2	mitochondrial malate dehydrogenase	36357	8.87	88	3	3 matches<br>1 sequences	*Solanum Lycopersicum*
6706	YP_009430460.1	ATP synthase subunit 1 (mitochondrion)	55581	5.93	258	6	3 matches<br>3 sequences	*Solanum Lycopersicum*
7301	XP_004232705.1	putative lactoylglutathione lyase-like	32953	5.95	338	15	3 matches<br>3 sequences	*Solanum Lycopersicum*
7601	XP_004250240.1	UTP--glucose-1-phosphate uridylyltransferase isoform X1	52014	5.384	158	5	2 matches<br>2 sequences	*Solanum Lycopersicum*
8601	XP_006365860.1	UTP--glucose-1-phosphate uridylyltransferase-like	52130	5.69	648	13	5 matches<br>5 sequences	*Solanum tuberosum*
8801	XP_004245731.1	hsp70-Hsp90 organizing protein 2	65410	5.99	85	2	1 matches<br>1 sequences	*Solanum Lycopersicum*
8901	XP_004253396.2	multicystatin, partial	85008	5.69		5	4 matches<br>2 sequences	*Solanum Lycopersicum*
