# Supplementary material for: Response mechanisms induced by exposure to high temperature in anthers from thermo-tolerant and thermo-sensitive tomato plants: A proteomic perspective
Source: PLoS One. 2018 Jul 19;13(7):e0201027. doi: 10.1371/journal.pone.0201027 (PMC6053223; doi:10.1371/journal.pone.0201027)
Supplement: S4 Table — Functional classification of the identified proteins and regulation of their abundance are reported. (PDF) [file pone.0201027.s007.pdf]

S4 Table. Functional classification of proteins potentially involved in the thermo-tolerance features and regulation of their abundance.

				HT/CC (fold change) [a)]		M82/SAL (fold change) [b)]	
ACCESSION NCBInr	PROTEIN NAME	SOL GENOMICS NETWORK ID GENE ID	PATHWAY OR GO TERMS	IA I SAL	IA II M82	IA III CC	IA IV HT
			**ENERGY METABOLISM**				
NP_001234080.1	enolase	Solyc09g009020.2 544068 PGH1, ER28	Glycolysis I-V(Plant Cytosol)	2.38	2.44	-2.35	-2.30
NP_001266254.2	glyceraldehyde 3-phosphate dehydrogenase	Solyc05g014470.2 101258368 GAPC2, GAPDH		1.50	2.88	-1.55	1.24
NP_001234293.2	succinyl-CoA ligase [ADP-forming] subunit beta, mitochondrial	Solyc06g083790.2 543863	TCA Cycle Variation III-IV Glyoxylate Cycle	5.44	3.26	1.06	-1.57
NP_001296789.1	pyruvate dehydrogenase E1 component subunit alpha, mitochondrial	Solyc05g006520.2 543639	Acetyl-Coa Biosynthesis	4.66	1.74	-1.03	-2.74
XP_004244101.1	dihydrolipoyllysine-residue succinyltransferase component of 2-oxoglutarate dehydrogenase complex 2, mitochondrial-like	Solyc07g064800.2 101268590	2-Ketoglutarate Dehydrogenase Complex	1.90	4.50	-1.87	1.26
			**AMINOACID METABOLISM**				
XP_004240034.1	glutamine synthetase-like	Solyc05g051250.2 101261030	Nitrate Reduction II-VI (Assimilatory) Ammonia Assimilation Cycle II Glutamine Biosynthesis	1.16	1.83	-13.59	-8.58
NP_001309987.1	glutamine synthetase cytosolic isozyme 1-1	Solyc04g014510.2 543756 gts1		1.75	2.44	-1.50	-1.04
NP_001310599.1	glutamine synthetase	Solyc01g080280.2 543998 GS2		1.86	2.59	-1.50	-1.06
NP_001233850.2	glutamate dehydrogenase	Solyc10g078550.1	Glutamate Biosynthesis/	1.62	3.74	-1.78	1.30

		544015<br>gdh1	Degradation				
NP_001296305.1	S-adenosylmethionine synthase 2	Solyc12g099000.1<br>101247506<br>SAM2	Phytosiderophore Biosynthesis<br>S-Adenosyl-L-Methionine Cycle/Biosynthesis	1.56	7.21	-1.94	2.38
NP_001234004.1	S-adenosylmethionine synthetase 3	Solyc09g008280.1<br>544302<br>SAM3		1.81	5.49	-1.77	1.71
			**OTHER METABOLISMS**				
XP_004230766.1	caffeoyl-CoA O-methyltransferase 6	Solyc01g107910.2<br>101260278	Esculetin Biosynthesis	-1.64	-2.48	1.59	1.05
NP_001318059.1	polyphenol oxidase F, chloroplastic	Solyc08g074630.1<br>101259064	Phenylpropanoid Biosynthesis	1.09	-3.22	3.63	1.03
NP_001296326.1	polyphenol oxidase B, chloroplastic;	Solyc08g074680.2<br>101258774<br>PPO		-1.55	-2.62	1.70	1.01
NP_001233847.1	carbonic anhydrase	Solyc02g067750.2<br>100147727<br>ca3	Cyanate Degradation	1.68	5.58	-1.92	1.73
NP_001233884.2	Leucine aminopeptidase 2, chloroplastic	Solyc00g187050.2<br>544277<br>LAPA2	Seed Germination Protein Turnover<br>Wound-Induced Proteolysis I	1.86	4.50	-1.92	1.26
			**FOLDING, SORTING AND DEGRADATION**				
XP_004247428.1	uncharacterized protein OsI_027940	Solyc09g075010.2<br>101249490	Chaperone Binding	4.22	1.80	1.25	-1.88
XP_004247810.1	chaperonin CPN60-2, mitochondrial	Solyc09g091180.2<br>101250927	ATP Binding	2.53	3.18	-1.94	-1.54
NP_001234183.1	plastid lipid associated protein CHRC	Solyc02g081170.2<br>778336<br>ChrC	Protein Binding	-2.62*	-2.21*	2.63*	3.11*
			**OTHER FUNCTIONS**				
NP_001266269.1	inducible plastid-lipid associated protein	Solyc07g064600.2<br>101248695	Endoribonuclease Activity	1.72	4.67	-1.89	1.53

		CHRDi					
AFJ93093.1	proteinase inhibitor II	Solyc03g020060.2	Serine-Type Endopeptidase Inhibitor Activity	-1.28	-1.94	2.28	1.50
XP_004235415.1	serine protease inhibitor 5-like	Solyc03g098760.1 101263388	Endopeptidase Inhibitor Activity	1.01	2.07	-5.06	-2.47
XP_004253396.2	multicystatin, partial	Solyc00g071180.2 543570	Cysteine-Type Endopeptidase Inhibitor Activity Cobalt Ion Binding	3.21*	2.39*	-1.60*	-2.16*
XP_010324012.1	stress-response A/B barrel domain-containing protein UP3-like	Solyc07g041490.1 101256396		-2.10	-3.47	2.50	1.52

a) Changes in protein levels are reported as the ratio between the normalized protein spot volume from *Saladette* and *M82* tomato anthers grown under high temperature and control conditions (V_{HT}/V_{CC}) for up-regulated proteins and as the negative reciprocal values ($-V_{HT}/V_{CC}$) for down-regulated proteins.

b) Changes in protein levels are reported as the ratio between the normalized protein spot volume from *M82* and *Saladette* tomato anthers grown under the same conditions (V_{M82}/V_{SAL}) for up-regulated proteins and as the negative reciprocal values ($- V_{M82}/V_{SAL}$) for down-regulated proteins.

* Average fold change for proteins contained in more than one spot has been calculated summing the normalized protein spot volume of all the spots containing the same protein and a fold change ≥ 2.0 has been considered significant.
